# Imbalanced Activation of Wnt-/β-Catenin-Signaling in Liver Endothelium Alters Normal Sinusoidal Differentiation

**DOI:** 10.3389/fphys.2021.722394

**Published:** 2021-09-29

**Authors:** Philipp-Sebastian Koch, Kajetan Sandorski, Joschka Heil, Christian D. Schmid, Sina W. Kürschner, Johannes Hoffmann, Manuel Winkler, Theresa Staniczek, Carolina de la Torre, Carsten Sticht, Kai Schledzewski, Makoto Mark Taketo, Felix A. Trogisch, Joerg Heineke, Cyrill Géraud, Sergij Goerdt, Victor Olsavszky

**Affiliations:** ^1^Department of Dermatology, Venereology and Allergology, University Medical Center and Medical Faculty Mannheim, Heidelberg University and Center of Excellence in Dermatology, Mannheim, Germany; ^2^European Center for Angioscience, Medical Faculty Mannheim, Heidelberg University, Mannheim, Germany; ^3^Next Generation Sequencing Core Facility, Medical Faculty Mannheim, Heidelberg University, Mannheim, Germany; ^4^Division of Experimental Therapeutics, Graduate School of Medicine, Kyoto University, Kyoto, Japan; ^5^Department of Cardiovascular Physiology, Medical Faculty Mannheim, Heidelberg University, Mannheim, Germany; ^6^Section of Clinical and Molecular Dermatology, Department of Dermatology, Venereology and Allergology, University Medical Center and Medical Faculty Mannheim, Heidelberg University, Mannheim, Germany

**Keywords:** mice, liver, liver sinusoidal endothelial cells, endothelial cells, triglycerides

## Abstract

Endothelial wingless-related integration site (Wnt)-/β-catenin signaling is a key regulator of the tightly sealed blood–brain barrier. In the hepatic vascular niche angiokine-mediated Wnt signaling was recently identified as an important regulator of hepatocyte function, including the determination of final adult liver size, liver regeneration, and metabolic liver zonation. Within the hepatic vasculature, the liver sinusoidal endothelial cells (LSECs) are morphologically unique and functionally specialized microvascular endothelial cells (ECs). Pathological changes of LSECs are involved in chronic liver diseases, hepatocarcinogenesis, and liver metastasis. To comprehensively analyze the effects of endothelial Wnt-/β-catenin signaling in the liver, we used endothelial subtype-specific *Clec4g-iCre* mice to generate hepatic ECs with overexpression of Ctnnb1. In the resultant *Clec4g-iCre*^*tg*/*wt*^*;Ctnnb1(Ex3)*^*fl*/*wt*^ (*Ctnnb1*^*OE*−*EC*^) mice, activation of endothelial Wnt-/β-catenin signaling resulted in sinusoidal transdifferentiation with disturbed endothelial zonation, that is, loss of midzonal LSEC marker lymphatic vessel endothelial hyaluronic acid receptor 1 (Lyve1) and enrichment of continuous EC genes, such as cluster of differentiation *(CD)34* and *Apln*. Notably, gene set enrichment analysis revealed overrepresentation of brain endothelial transcripts. Activation of endothelial Wnt-/β-catenin signaling did not induce liver fibrosis or alter metabolic liver zonation, but *Ctnnb1*^*OE*−*EC*^ mice exhibited significantly increased plasma triglyceride concentrations, while liver lipid content was slightly reduced. Ctnnb1 overexpression in arterial ECs of the heart has been reported previously to cause cardiomyopathy. As *Clec4g-iCre* is active in a subset of cardiac ECs, it was not unexpected that *Ctnnb1*^*OE*−*EC*^ mice showed reduced overall survival and cardiac dysfunction. Altogether, balanced endothelial Wnt-/β-catenin signaling in the liver is required for normal LSEC differentiation and for maintenance of normal plasma triglyceride levels.

## Introduction

Liver sinusoidal endothelial cells (LSECs) lining the hepatic sinusoids are a prime example for organ-specific endothelial differentiation. They belong to the group of discontinuous ECs characterized by an incomplete basement membrane and the presence of large fenestrations without diaphragm. LSECs exhibit unique molecular, phenotypic, and functional features and are known to instruct the hepatic vascular niche by cellular interactions and the secretion of paracrine-acting factors called angiokines (Nolan et al., 2013; Augustin and Koh, 2017). For example, LSECs were identified to control liver regeneration by angiocrine wingless-related integration site 2 (Wnt2) and hepatocyte growth factor (Hgf) signaling (Ding et al., 2010; Cao et al., 2017; Zhang et al., 2020). Other highly specialized functions include immunological processes, such as tolerance and defense mechanisms and the clearance of noxious factors from the circulation by a repertoire of scavenger receptors (Schledzewski et al., 2011; Wittlich et al., 2017; Shetty et al., 2018). Interestingly, pathological changes of these highly specialized ECs were shown to contribute to severe liver diseases ranging from steatohepatitis to liver cirrhosis and from hepatocarcinogenesis to liver metastasis (Kostallari and Shah, 2016). During disease processes, LSECs are known to transdifferentiate toward a capillary phenotype revealing a loss of fenestrations and formation of a basement membrane, which is termed “sinusoidal capillarization,” thereby aggravating disease progression (Schaffner and Popper, 1963; Lalor et al., 2006). However, the molecular and signaling mechanisms driving sinusoidal capillarization still await detailed analysis.

Notably, recent work by our group could identify the transcription factor GATA-binding factor 4 (GATA4) as a molecular master regulator for LSEC differentiation during liver development and in liver homeostasis. LSEC-restricted deletion of *Gata4* was shown to cause transformation of discontinuous liver sinusoids into continuous capillaries. This sinusoidal transformation in the fetal liver inhibited homing of hematopoietic stem and progenitor cells into the fetal liver resulting in fatal anemia (Geraud et al., 2017), while *Gata4* deletion in the mature vasculature caused hepatopathy and perisinusoidal liver fibrosis (Winkler et al., 2021). A special form of liver sinusoidal capillarization was also demonstrated when endothelial Notch signaling was enhanced resulting in a partial loss of LSEC-specific markers and increased the expression of continuous endothelial cell (CEC) markers; however, lacking the formation of a solid basement membrane or liver fibrogenesis (Wohlfeil et al., 2019).

Another highly conserved transduction pathway known to be involved in several important biological processes, such as liver development, vascular and hepatic differentiation, and tissue homeostasis is the Wnt-/β-catenin signaling pathway (Decaens et al., 2008; Daneman et al., 2009; Wild et al., 2020). In the liver vasculature, Wnt2 was identified as an LSEC-associated molecule with autocrine growth effects (Klein et al., 2008; Geraud et al., 2010), and also as an angiocrine regulator of liver regeneration (Ding et al., 2010). In the meantime, angiocrine Wnt signaling in the liver has become even more important. Hepatic endothelial cells (ECs) not only express Wnt2, they also express Wnt9b, Wnt ligand secretion mediator (Wls), and Wnt potentiator R-Spondin 3 (Geraud et al., 2010; Rocha et al., 2015). These factors are indispensable for the formation of a Wnt-dependent pericentral hepatocyte subpopulation. Loss of this Wnt-signaling machinery results in decreased liver size, perturbations of liver zonation, metabolic maturation, and impaired liver regeneration capacity (Rocha et al., 2015; Wang et al., 2015; Planas-Paz et al., 2016; Leibing et al., 2018; Preziosi et al., 2018).

Considering EC morphogenesis and specification, Wnt signaling emerged as a major contributor in the past decades (Choi et al., 2012). ECs not only express intracellular Wnt molecules and their corresponding Frizzled receptors, but also β-catenin-dependent transcription factors (Masckauchan et al., 2005; Deb, 2014). Hereby, it was shown that β-catenin induces arterialization and loss of venous fate of the embryonic vasculature during development (Duarte et al., 2004; Corada et al., 2010). Moreover, in vascular beds of the central nervous system Wnt-signaling is a key regulator of the integrity of the highly sealed blood–brain barrier (BBB) by controlling the formation of tight junction (TJ) molecules and solute transporters (Liebner et al., 2008; Zhou et al., 2014; Tran et al., 2016; Profaci et al., 2020). LSECs, on the other hand, do not typically express TJs, since permeability and cell trafficking are facilitated by open fenestrations (Geraud et al., 2012). Constitutive activation of β-catenin in the highly permeable ECs of the circumventricular organs in the brain resulted in the expression of BBB markers and downregulation of non-BBB vasculature markers (Benz et al., 2019; Wang et al., 2019). As our study and other previous studies have shown that Wnt2 is a LSEC-specific growth and differentiation factor required for liver regeneration and that autocrine Wnt/β-catenin signaling cross-stimulates the angiogenetic vascular endothelial growth factor receptor 2 pathway, we hypothesized that unbalanced canonical endothelial Wnt signaling in the liver might also impair LSEC differentiation and LSEC-mediated liver function. To test this hypothesis, we generated a novel mouse line with constitutive β-catenin overactivation in LSECs by crossing *Ctnnb1-Ex3*^*fl*/*wt*^ with EC subtype-specific *Clec4g-iCre*^*tg*/*wt*^ mice (Wohlfeil et al., 2019).

## Materials and Methods

### Animals

To generate endothelial subtype-specific Ctnnb1 gain-of-function (GOF) mice (*Ctnnb1*^*OE*−*EC*^), *Clec4g-iCre*^*tg*/*wt*^ (*Tg(Clec4g-icre*)*1.1Sgoe* (Wohlfeil et al., 2019) were crossed with *Ctnnb1(Ex3)*^*fl*/*wt*^ (*Ctnnb1*^*tm1Mmt*^) (Harada et al., 1999) mice. Specificity of Cre-activity was analyzed in crosses of *Clec4g-iCre*^*tg*/*wt*^ transgenic mice with *R26YFP* (*B6.129X1-Gt(ROSA)26Sortm1(EYFP)Cos/J*) [JAX 006148] (Srinivas et al., 2001) reporter animals. All animals were housed under specific pathogen-free conditions in an animal facility (Heidelberg University). Animal experiments were performed in accordance with Federal Animal Regulations and were institutionally approved by the district government Karlsruhe and performed under institutional guidelines. Mice were sacrificed by cervical dislocation. Liver, heart, kidney, lung, spleen, brain, and intestine weights were measured, and tissue samples were either embedded in the optimum cutting temperature compound (Sakura, Alphen aan den Rijn, The Netherlands) and frozen in liquid nitrogen or fixed in 4% paraformaldehyde at 4°C.

### Isolation of Primary Murine LSECs

Livers, pooled from two mice, were perfused *in situ* via the portal vein with a 0.05% collagenase containing amino acid/saccharide calcium-deprived medium (C2674, Sigma–Aldrich, Taufkirchen, Germany), dissected, mechanically minced, digested at 38°C in a collagenase/Gey's balanced salt solution (G9779, Sigma–Aldrich) and filtered through a 250 μm mesh. Cells were separated by a 35% Nycodenz (1002424, Axis-Shield, Alere Technologies, Oslo, Norway) gradient. Next, LSECs were isolated by magnetic-activated cell sorting using anti-CD146 MicroBeads (ME-9F1, 130-092-007, Miltenyi Biotech, Bergisch Gladbach, Germany) according to the instructions of the manufacturers.

### Quantitative Reverse-Transcription PCR

RNA was extracted from primary ECs using EZNA Total-RNA-Kit I (OMEGA Biotec, Norcross, GA, United States). Complementary DNA (cDNA) was synthesized with RevertAid H-Minus M-MuLV Reverse Transcriptase (ThermoScientific, Waltham, MA, United States). Quantitative PCR was performed on a qTOWER 3 G touch thermal cycler (Analytik Jena) using innuMIX qPCR SyGreen Sensitive (845-AS-1310200, Analytik Jena, Jena, Germany). Normalized expression values were calculated using the Pfaffl method considering amplification efficiency values determined by standard curves (Pfaffl, 2001).

### RNA *in situ* Hybridization

Liver tissue was sectioned at 4 μm. RNA *in situ* hybridization (ISH) was conducted using RNAscope 2.5 HD Red (322350, Advanced Cell Diagnostics, Newark, CA, United States) kits with mouse-specific probes against the positive control mouse *Ppib* (*Cyclophilin B*) gene, *Mus musculus* (Mm)-*Bmp2*-E3-Channel 1 (1545–2347 NM_007553.3), Mm-*Hgf*-Channel 1 (1120–2030 NM_010427.4), Mm-*Wnt2*-Channel 1 (857–2086 NM_023653.5), Mm-*Wnt9b*-Channel 1 (706–1637 NM_011719.4), Mm-*Stab1*-Channel 1 (488-1320 NM_138672.2), and Mm-*Stab2*-Channel 1 (4249–5075 NM_138673.2) according to the protocols of the manufacturer.

### Histology and Immunofluorescence

Tissue samples fixed by 4% paraformaldehyde at room temperature for 48–72 h, were subsequently transferred into phosphate-buffered saline (PBS), dehydrated in a graded alcohol series, and embedded in paraffin. Paraffin-embedded tissues were sectioned in 4 μm. For hematoxylin & eosin (H&E), periodic acid–Schiff (PAS), Oil Red O (ORO), Prussian blue, and Sirius red staining, samples were processed according to the standard protocols provided by the manufacturer. For immunofluorescence (IF), cryosections (7 μm) were air-dried, fixed in 4% paraformaldehyde (PFA) or acetone, rehydrated in PBS (A0964.9050, VWR International, Radnor, PA, United States) and blocked in 5% donkey serum (017-000-121, Dianova, Hamburg, Germany) in PBS for 30 min. Primary antibodies were incubated overnight at 4°C. Sections were washed three times in PBS before incubation with appropriate Alexa Fluor–coupled secondary antibodies for 45 min at room temperature. Paraffin-embedded sections were baked at 60°C overnight, after which they were deparaffinized with xylol and rehydrated using ethanol in decreasing concentrations. Antigen retrieval of tissue sections was carried out with epitope retrieval solution (Zytomed Systems, Berlin, Germany) at either pH 6, 8, or 9. Primary antibody was incubated for 2 h at room temperature or overnight at 4°C. Sections were washed three times in PBS before incubation of appropriate secondary antibodies for 1 h at room temperature. Nuclei were counterstained with 4′,6-diamidin-2-phenylindol (DAPI) (D1306, Thermo Fisher Scientific, Waltham, MA, United States). Finally, sections were thoroughly washed in PBS before mounting with Dako fluorescent mounting medium (Dako, Agilent technologies, Santa Clara, CA, United States). Sections were photographed with ECLIPSE Ci microscope (Nikon, Alzenau, Bavaria, Germany) or ECLIPSE Ni-E microscope (Nikon). Immunofluorescence images were acquired in a sequential mode as a series of *z*-axis images and processed with NIS-Elements AR 5.02 (Nikon Instruments, Tokyo, Japan) and ImageJ 1.52e software (NIH, Bethesda, MD, United States). Using NIS-Elements AR 5.02, images were background corrected (rolling ball 7.5 pixels), deconvoluted, and focused to one plane.

For the quantification of IF images, three representative areas per sample were chosen. For each image, binary masks of the representative channels were created using automated threshold functions (Otsu, MaxEntropy) in ImageJ. The resulting binary masks were quantified for the area, or the number of particles (10-infinite pixels) using ImageJ functions “Measure” and “Analyze Particles.” The “Mean gray value” represents the sum of the gray values of all the pixels within the selected images divided by the number of all pixels. For Ki-67 quantification, only Ki-67 staining was included that overlaid with DAPI-positive nucleus staining to exclude unspecific signal. To this end, we used the “Image Calculator” in ImageJ with “AND” as operator for Ki-67 and DAPI channels. For quantification of RNA ISH or ORO images, three representative areas per sample were chosen. RGB images were split into separate channels corresponding to three determined colors by using the “Color deconvolution” command in ImageJ. The images displaying the region of interest were further processed by setting color thresholds. Finally, the area of particles (>30 pixels) was measured, analyzed, and calculated in percentage (%) of the whole image area.

### Antibodies

Primary antibodies: rat anti-Endomucin (14-5851-82, eBioscience, San Diego, CA, United States), goat anti-Lyve1 (AF2125, R&D Systems, Minneapolis, MN, United States), rat anti-mouse/human GATA-4 (14-9980-82, Thermo Fisher Scientific), rat anti-mouse CD68 (137002, BioLegend, San Diego, CA, United States), rabbit anti-Desmin (ab15200, Abcam, Cambridge, Cambs., UK), rabbit anti-glutamine synthetase (G2781, Sigma–Aldrich, Taufkirchen, Bavaria, Germany), goat anti-arginase I (sc-18351, Santa Cruz Biotechnology, Dallas, TX, United States), rat anti-Ki67 (14-5698-82, eBioscience), polyclonal rabbit anti- green fluorescent protein/yellow fluorescent protein (YFP) (A11122, Molecular Probes, Eugene, OR, United States), rat anti-CD31 (102502, BioLegend), SMA-antibody (ab5694, Abcam), goat anti-CD32b (AF1460, R&D Systems), rabbit anti-Collagen type I (R1038, Acris, Hiddenhausen, North Rhine-Westphalia, Germany), rabbit anti-Collagen type III alpha 1 chain (R1040, Acris), rabbit anti-Collagen IV (GTX19808, Genetex, Irvine, CA, United States), rabbit anti-Cyp2E1 (HPA009128, Sigma–Aldrich), rabbit anti-Claudin 5 (34-1600, Thermo Fisher Scientific), goat anti-Podocalyxin (AF1556, R&D Systems), rabbit anti-Cav1 (N-20, Santa Cruz Biotechnology), rabbit anti- intracellular adhesion molecule 1 (ICAM1) (10020-1-AP, Proteintech, Rosemont, IL, United States), goat anti-mouse vascular endothelial (VE)-cadherin (AF1002, R&D Systems), goat anti- vascular cell adhesion molecule (VCAM)-1/CD106 (AF643, R&D Systems). Secondary antibodies: Alexa-Fluor 488, Alexa-Fluor 647, and cyanine 3-conjugated secondary antibodies were purchased from Dianova (Hamburg, Germany).

### Microarray Processing and Statistical Analysis

Gene expression profiling was performed using arrays MoGene-2_0-st from Affymetrix (Santa Clara, CA, United States). Biotinylated antisense cDNA and arrays hybridization were performed according to the recommendations of the manufacturer using the GeneChip WT Plus Reagent Kit and the GeneChip Hybridization, Wash and Stain Kit (both from Thermo Fisher Scientific). A Custom CDF Version 22 with ENTREZ-based gene definitions was used to annotate the arrays. The raw fluorescence intensity was robust multiarray analysis background corrected and values were normalized applying quantile normalization. Differential gene expression was analyzed with the one-way-ANOVA, using a commercial software package SAS JMP15 Genomics, version10, from SAS (SAS Institute, Cary, NC, United States). A false-positive rate of *a* = 0.05 with FDR correction was taken as the level of significance. To determine whether defined lists (or sets) of genes exhibit a statistically significant bias in their distribution, we performed a gene set enrichment analysis (GSEA). GSEA (Subramanian et al., 2005) was carried out using R 3.6.1. clusterProfiler 3.12.0 (Yu et al., 2012), fgsea 1.10.0 (Korotkevich et al., 2021), the molecular signatures database (MSigDB) v6.2 hallmark gene set collection (Liberzon et al., 2015), and self-defined gene lists were used. Gene lists for LSECs and CECs were used as previously described (Winkler et al., 2021). The gene set for brain ECs was defined using published single-cell RNA seq data (Sabbagh et al., 2018). Inclusion criteria were fold change ≥ 2 for brain *vs*. liver ECs and at least 10 transcripts per million in liver ECs to exclude less-expressed genes. Overrepresentation analysis (ORA) of Gene Ontology terms was performed with the enrichR (Chen et al., 2013) package in R 3.6.1 for all significantly regulated genes. Heatmaps were created with the ComplexHeatmap package (Gu et al., 2016).

The raw and normalized gene expression profiling data have been deposited in the NCBI Gene Expression Omnibus and are accessible through GEO Series accession number GSE175777 (https://www.ncbi.nlm.nih.gov/geo/query/acc.cgi?acc=GSE175777).

### Blood Parameters

Serum was analyzed for the following routine parameters: alanine aminotransferase (ALA), aspartate aminotransferase (AST), and glutamate dehydrogenase (GLDH), cholesterol, triglycerides, glucose, and total protein (Roche cobas c 311 analyser, Roche Diagnostics, Basel, Switzerland).

### Hepatic Triglycerides

Snap frozen liver tissue (100 mg) was homogenized in 5% NP-40 solution (74385, Merck) and heated for 5 min in a shaking dry incubator (ThermoMixer C, Eppendorf, Hamburg, Germany) at 80–100°C. After cooling to room temperature, the heating was repeated in order to solubilize all triglycerides. After centrifugation for 2 min at top speed (Centrifuge 5417 R, Eppendorf) the supernatant was diluted 10-fold in distilled water and used to determine the triglyceride content based on the protocol of the Triglyceride Quantification Colorimetric/Fluorometric Kit manufacturer (K622, BioVision, Mountain View, CA, United States).

### Transthoracic Echocardiography

For echocardiography, mice were anesthetized with 0.5–1.0% isoflurane and placed on a heating pad to maintain body temperature. Non-invasive, echocardiographic parameters were recorded with a linear 50 MHz transducer (Vevo 3100 system with MX700 transducer, Visualsonics, Toronto, Canada) in parasternal long-axis B- & M-mode, and measured post-processing, which comprised heart rate, left ventricle (LV) posterior and anterior wall thickness, and LV internal diameter at both end-systole and end-diastole. From that, LV volume, LV ejection fraction, and cardiac output were calculated with the Vevo Workstation 5.5.0 and the integrated cardiac measurement package.

### Statistics

Statistical analysis was performed with SigmaPlot 11 Software (Systat Software GmbH, Germany). For pairwise comparisons, the *t*-test was used when normality was proved. Differences between data sets with *p* < 0.05 were considered statistically significant. Data are presented as means with error bars indicating standard error.

## Results

### Generation and Characterization of Adult β-Catenin-Overactivated HEC Mice

**Figure 1:**
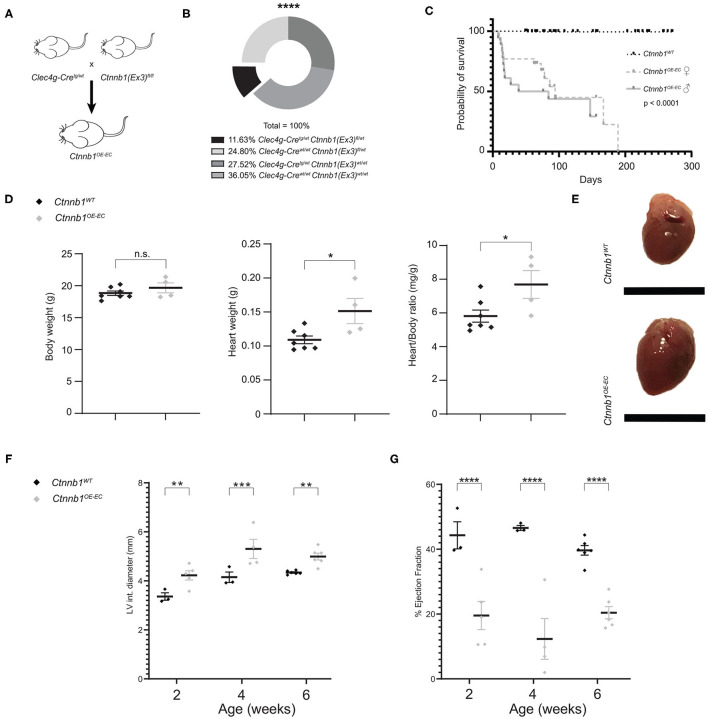
*Ctnnb1*^*OE*−*EC*^ mice have a low survival probability and suffer from cardiac dysfunction **(A)** *Ctnnb1*^*OE*−*EC*^ mice [*Clec4g-iCre*^*tg*/*wt*^;*Ctnnb1(Ex3)*^*fl*/*wt*^] were generated by crossing *Clec4g-iCre*^*tg*/*wt*^ with *Ctnnb1(Ex3)*^*fl*/*fl*^ mice. **(B)** Mendelian frequency of *Clec4g-iCre*^*tg*/*wt*^;*Ctnnb1(Ex3)*^*fl*/*wt*^ mice. ^****^p < 0.0001. **(C)** Kaplan–Meier survival curves for control (*Ctnnb1*^*WT*^) and *Ctnnb1*^*OE*−*EC*^ mice. The probability of survival is shown for *Ctnnb1*^*OE*−*EC*^ female (*n* = 26) vs. *Ctnnb1*^*OE*−*SEC*^ male (*n* = 18) vs. *Ctnnb1*^*WT*^ mice (*n* > 65). **(D)** Body weight, heart weight, heart-to-body weight ratio of 2- to 3-month-old *Ctnnb1*^*WT*^ and *Ctnnb1*^*OE*−*EC*^ mice (female, *n* ≥ 4). Results are represented as mean ± SEM. ns, not significant; ^*^p < 0.05. **(E)** Macroscopic heart images of 3-month-old *Ctnnb1*^*WT*^ and *Ctnnb1*^*OE*−*EC*^ mice (female, *n* = 4). Scale bar 1 cm. **(F)** Left ventricle (LV) interior diameter and **(G)** ejection fraction as determined by echocardiography of 2-, 4-, and 6-week-old *Ctnnb1*^*WT*^ and *Ctnnb1*^*OE*−*EC*^ mice (*n* ≥ 3). Results are represented as mean ± SEM. ^**^p < 0.01; ^***^p < 0.001; ^****^p < 0.0001.

EC subtype-specific *Clec4g-iCre* mice (Wohlfeil et al., 2019) were used to generate mice with Ctnnb1 GOF in LSECs (Figure 1A). *Ctnnb1*^*OE*−*EC*^ (*Clec4g-iCre*^*tg*/*wt*^*;Ctnnb1(Ex3)*^*fl*/*wt*^) mice were viable but were born at a lower Mendelian frequency than expected (Figure 1B) and suffered from a reduced overall survival rate (Figure 1C). While bodyweight was not altered, heart weight as well as heart weight/body weight ratio were significantly increased in *Ctnnb1*^*OE*−*EC*^ mice (Figures 1D,E). As Cre-activity was previously described in ECs of the heart in *Clec4g-iCre* mice (Wohlfeil et al., 2019), a comprehensive analysis of *Clec4g-iCre;R26YFP* reporter mice was performed for this organ. Reporter activity was present in CD31^+^ ECs of the heart (Supplementary Figure 1A). Specifically, YFP positivity was observed in the endocardium, including endomucin (Emcn)^+^ endocardial trabeculae (Rhee et al., 2018) as well as in CD31^+^ αSMA^+^ coronary veins and arteries (Zhang et al., 2005) (Supplementary Figures 1A,B). In contrast, LYVE1^+^ lymphatic vessels were YFP negative (Supplementary Figure 1B). In *Ctnnb1*^*OE*−*EC*^ mice, echocardiography revealed progressive cardiac dysfunction, which is comparable to the phenotype obtained after β-catenin GOF mutation in arterial ECs of the heart by using *Bmx-CreER*^T2^ transgenic mice (Nakagawa et al., 2016). *Ctnnb1*^*OE*−*EC*^ mice displayed increased end-diastolic left ventricle internal diameters and volumes (Figure 1F; Supplementary Figure 2A) and a reduction in wall thickness of the left ventricle (Supplementary Figures 2B,C). The ejection fraction was significantly reduced starting with 2 weeks of age (Figure 1G), whereas the cardiac output was first reduced starting with 4 weeks of age (Supplementary Figure 2D). A routine histochemical staining of internal organs such as the kidneys, lungs, spleen, brain, and intestine were gross morphologically unremarkable (Supplementary Figure 3).

**Figure 2:**
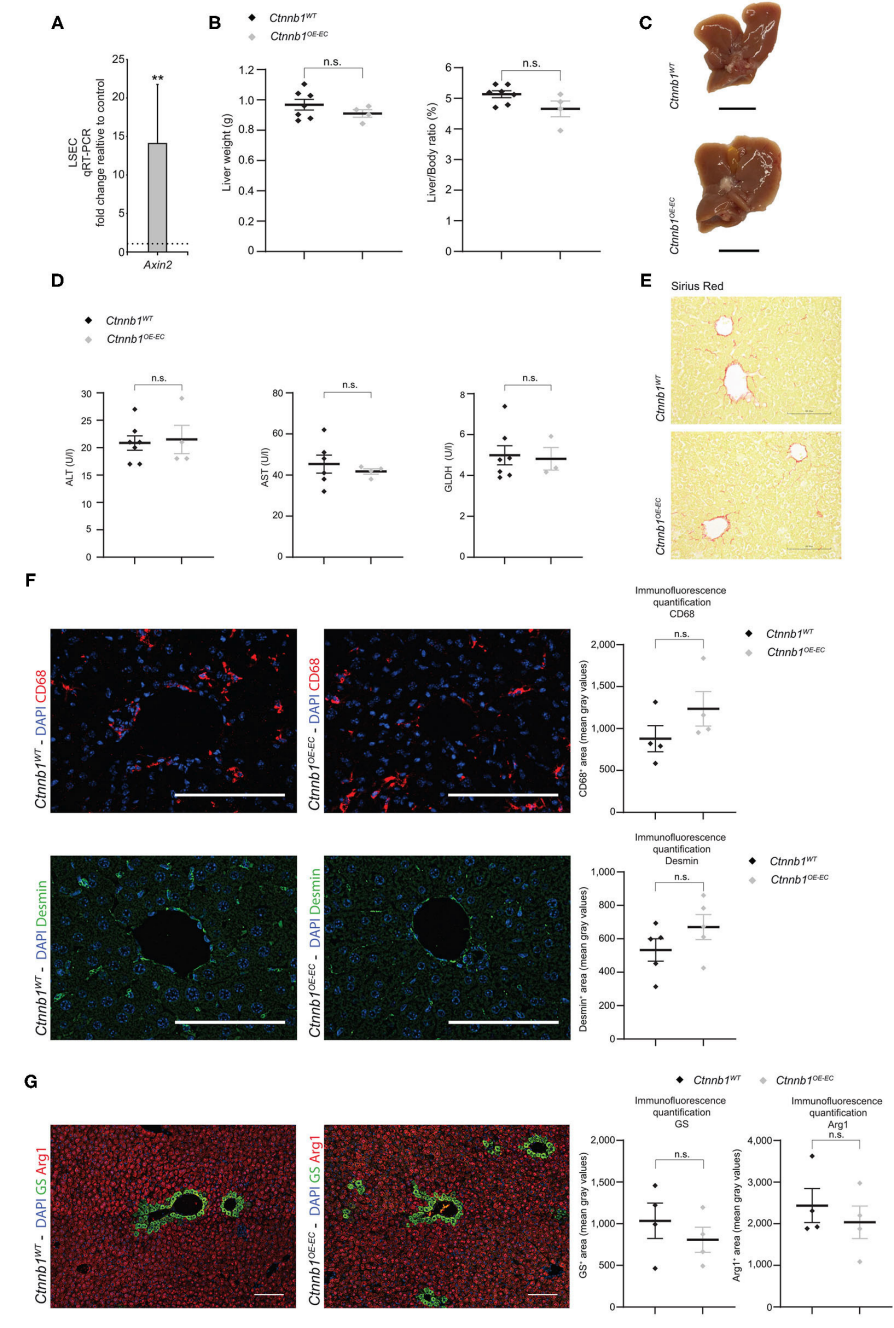
Hepatic endothelial *Ctnnb1* overactivation does not lead to hepatopathy and fibrosis. **(A)** qRT-PCR for axis inhibition protein 2 (*Axin2*) of cDNA from freshly isolated LSECs of 2-months-old *Ctnnb1*^*OE*−*EC*^ mice compared to corresponding *Ctnnb1*^*WT*^ controls (*n* = 3). β-Actin was used as housekeeping gene. ^**^p < 0.01. **(B)** Liver weight, liver-to-body weight ratio of 2- to 3-month-old *Ctnnb1*^*WT*^ and *Ctnnb1*^*OE*−*EC*^ mice (female, *n* ≥ 4). Results are represented as mean ± SEM. ns, not significant. **(C)** Macroscopic liver images of 3-month-old *Ctnnb1*^*WT*^ and *Ctnnb1*^*OE*−*EC*^ mice (female, *n* = 4). Scale bar 1 cm. **(D)** Liver enzymes [aspartate aminotransferase (AST), alanine aminotransferase (ALT), and glutamate dehydrogenase (GLDH)] in serum of 2- to 3-month-old female *Ctnnb1*^*WT*^ and *Ctnnb1*^*OE*−*EC*^ mice (*n* ≥ 3). Results are represented as mean ± SEM. n.s., not significant. **(E)** Sirius red staining of liver sections of 2- to 3-month-old male *Ctnnb1*^*WT*^ and *Ctnnb1*^*OE*−*EC*^ mice (*n* = 4). Scale bar 100 μm. **(F)** Immunofluorescence (IF) staining of DAPI, CD68 and Desmin, and CD68 and Desmin quantification in the liver of 2- to 3-month-old female *Ctnnb1*^*WT*^ and *Ctnnb1*^*OE*−*EC*^ mice (*n* ≥ 4). Scale bar 100 μm. Results are represented as mean ± SEM. ns, not significant. **(G)** IF staining of DAPI, glutamine synthetase (GS) and arginase (Arg1), and GS and Arg1 quantification in the liver of 2- to 3-month-old *Ctnnb1*^*WT*^ and *Ctnnb1*^*OE*−*EC*^ mice (*n* = 4). Scale bar 100 μm. Results are represented as mean ± SEM. ns, not significant.

**Figure 3:**
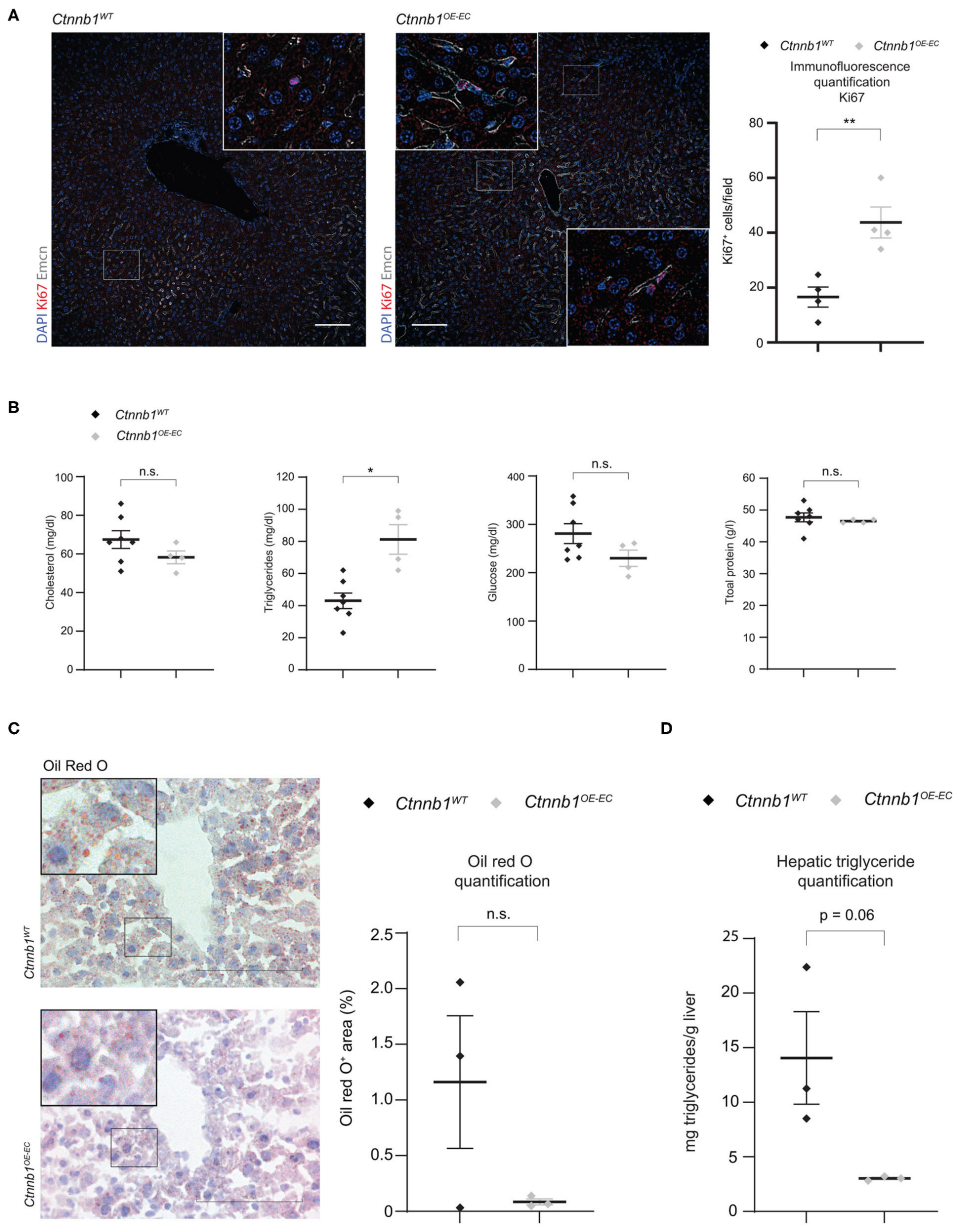
*Ctnnb1*^*OE*−*EC*^ mice display enhanced hepatic endothelial cell proliferation, serum hypertriglyceridemia, and decreased lipid accumulation in the liver. **(A)** IF staining of DAPI, Ki67, and Emcn, and Ki67 quantification in the liver of 2- to 3-month-old female *Ctnnb1*^*WT*^ and *Ctnnb1*^*OE*−*EC*^ mice (*n* = 3). Scale bar 100 μm. Results are represented as mean ± SEM. ^**^p < 0.01. **(B)** Serum levels of cholesterol, triglycerides, and glucose in 2- to 3-month-old *Ctnnb1*^*WT*^ and *Ctnnb1*^*OE*−*EC*^ mice (female, *n* ≥ 5). Results are represented as mean ± SEM. ^**^p < 0.01. **(C)** Oil Red O (ORO) staining and quantification of livers of 3-month-old female *Ctnnb1*^*WT*^ and *Ctnnb1*^*OE*−*EC*^ mice (*n* = 3). Scale bar 100 μm. Results are represented as mean ± SEM. ns, not significant. **(D)** Hepatic triglyceride concentration of murine liver tissue of 3-month-old female *Ctnnb1*^*WT*^ and *Ctnnb1*^*OE*−*EC*^ mice (*n* = 3). ^*^p < 0.05.

Endothelial β-catenin overactivation in the liver was confirmed by quantitative reverse-transcription PCR (qRT-PCR), which showed significantly elevated expression of Wnt-/β-catenin downstream target gene axis inhibition protein 2 (*Axin2*) in isolated LSECs from *Ctnnb1*^*OE*−*EC*^ mice (Figure 2A). Liver size, liver weight, and liver/body weight ratio were not significantly altered in *Ctnnb1*^*OE*−*EC*^ mice (Figures 2B,C). Basic liver function tests did not show elevated levels of ALT, AST, and GLDH (Figure 2D). Upon Sirius red staining, no signs of fibrosis were present in the *Ctnnb1*^*OE*−*EC*^ livers (Figure 2E). In line with the absence of collagen deposition upon Sirius red staining, no changes in perisinusoidal collagen I, III, or basement membrane collagen IV deposition were seen (Supplementary Figure 4A). Additionally, no obvious alterations were seen in livers of *Ctnnb1*^*OE*−*EC*^ mice upon H&E histology, PAS, and Prussian blue staining (Supplementary Figure 4B). Likewise, Kupffer cells or hepatic stellate cells (HSC) were not altered in quantity, as analyzed by IF for CD68 or Desmin, respectively (Figure 2F). Co-IF of marker proteins for metabolic liver zonation revealed no changes in zonated expression of Glul/GS and Cyp2E1 in pericentral or Arg1 in periportal and midlobular hepatocytes (Figure 2G; Supplementary Figure 4C). Notably, there was a significant increase in the Ki67-positivity in ECs from *Ctnnb1*^*OE*−*EC*^ livers, while the proliferation of hepatocytes did not show changes (Figure 3A).

Recently, we could demonstrate that EC-derived Wnt signaling controls metabolic liver zonation and alters lipid metabolism (Leibing et al., 2018). Although metabolic liver zonation was not affected by β-catenin GOF mutation, we performed a comprehensive metabolic screening including serum parameters such as total protein, cholesterol, triglycerides, and glucose (Figure 3B). Interestingly, *Ctnnb1*^*OE*−*EC*^ mice showed significantly increased serum levels of triglycerides (Figure 3B). Liver tissue of *Ctnnb1*^*OE*−*EC*^ mice showed slightly reduced lipid storage upon Oil Red O staining (Figure 3C) and a tendency of decreased levels of hepatic triglycerides as measured by a colorimetric assay (Figure 3D).

### Hepatic Endothelial β-Catenin GOF Mutation Causes Molecular Transdifferentiation of LSECs

**Figure 4:**
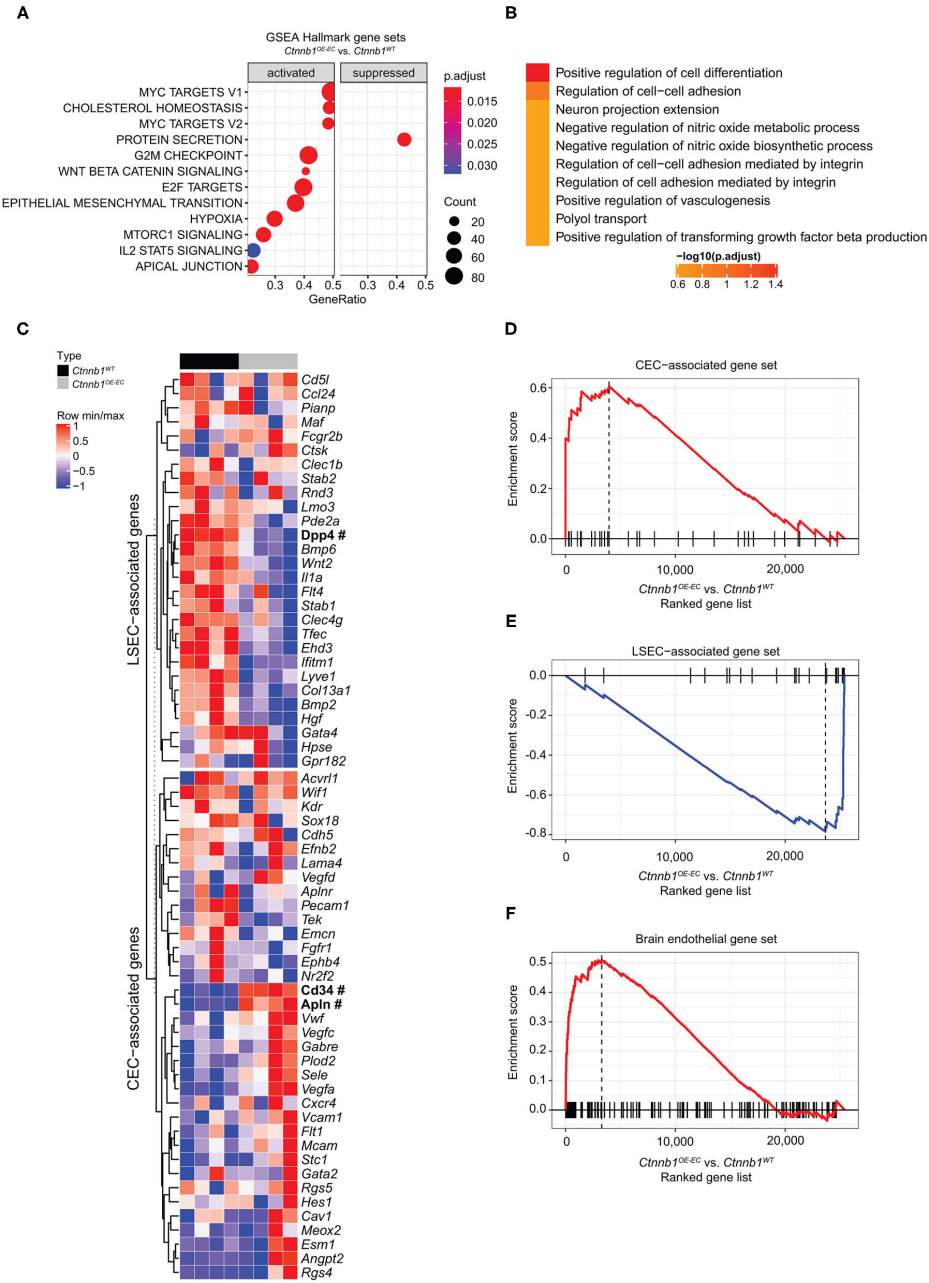
Hepatic endothelial *Ctnnb1* overactivation causes sinusoidal transdifferentiation. **(A)** Gene Set Enrichment Analysis-Kyoto Encyclopedia of Genes and Genomes (GSEA-KEGG) pathway alterations analyzed using MSigDB hallmark gene sets in freshly isolated LSECs from 2-month-old female *Ctnnb1*^*WT*^ and *Ctnnb1*^*OE*−*EC*^ (*n* = 4). **(B)** Overrepresentation analysis of gene ontology “biological processes” library. **(C)** Heat map of the liver sinusoidal endothelial cell (LSEC)- and continuous endothelial cell (CEC)-associated genes. Selected genes are shown for isolated LSECs from *Ctnnb1*^*WT*^ (black) and *Ctnnb1*^*OE*−*EC*^ mice (gray). Significant samples are written in bold and marked with # (*n* = 4 samples per group). The heat map color represents the mean and maximum values for each gene. The intensity scale of the standardized expression values ranges from dark blue (low expression) to dark red (high expression). Enrichment plots of **(D)** LSEC-associated (*p* = 0.0023; NES = −2.43) and **(E)** CEC-associated (*p* = 0.0023; NES = 2.04) genes (*n* = 4). **(F)** Enrichment plots of brain endothelial genes (*p* = 0.0001; NES = 2.16) (*n* = 4).

To identify β-catenin-dependent molecular alterations in LSECs, we performed comprehensive Affymetrix DNA microarray gene expression profiling of isolated primary LSECs from *Ctnnb1*^*WT*^ control and *Ctnnb1*^*OE*−*EC*^ animals. β-catenin GOF mutation in LSECs resulted in the significant dysregulation of 128 genes as compared to control LSECs (Table 1). GSEA of LSECs isolated from *Ctnnb1*^*WT*^ control and *Ctnnb1*^*OE*−*EC*^ animals revealed significant pathway alterations in the Hallmark gene sets. Among the most regulated gene sets, we found “Myc targets V1 and V2” and “Cholesterol homeostasis” (Figure 4A) followed by “G2M Checkpoint” and “E2F targets.” Furthermore, GSEA confirmed the activation of Wnt/β-catenin signaling in β-catenin GOF mutation in LSECs (Figure 4A). Overrepresentation analysis (ORA) of the significantly dysregulated genes by using Enrichr revealed significant alterations in the gene ontology (GO) biological processes 2018 library (Figure 4B). “Positive regulation of cell differentiation” was identified as the most significant GO term in LSECs with β-catenin GOF mutation (Figure 4B).

**Table 1:** Differentially expressed genes (DEGs) in *Ctnnb1*^*OE*−*EC*^-LSECs compared to wild-type controls.

**Gene symbol**	**Gene title**	**Fold change** ***Ctnnb1***^***OE***^**−**^***EC***^ > *Ctnnb1*^***WT***^	**Adjusted p-value for Diff of genotype =** ***Ctnnb1***^***OE***^**−**^***EC***^**–*Ctnnb1***^***WT***^
*Slc35f2*	Solute carrier family 35, member F2	35.13809	0.000397
*Apln*	Apelin	10.30015045	0.016367
*Susd4*	Sushi domain containing 4	9.257366289	0.007219
*Csf2rb2*	Colony stimulating factor 2 receptor, beta 2, low-affinity (granulocyte-macrophage)	8.781086232	0.041067
*Cd34*	CD34 antigen	8.598926088	0.001273
*Selp*	Selectin, platelet	8.373297581	0.037503
*Lypd6*	LY6/PLAUR domain containing 6	8.152209698	0.004191
*Glp1r*	Glucagon-like peptide 1 receptor	7.081854471	0.041067
*Myo1b*	Myosin IB	6.942870328	0.023473
*St8sia2*	ST8 alpha-N-acetyl-neuraminide alpha-2,8-sialyltransferase 2	5.709992179	0.022697
*Hunk*	Hormonally upregulated Neu-associated kinase	5.184396379	0.045899
*St8sia4*	ST8 alpha-N-acetyl-neuraminide alpha-2,8-sialyltransferase 4	4.998737335	0.0049
*Atp10a*	ATPase, class V, type 10A	4.856467229	0.012795
*Tcf7*	Transcription factor 7, T cell specific	4.800597937	0.013887
*Mal*	Myelin and lymphocyte protein, T cell differentiation protein	4.715053595	0.025665
*Ptgis*	Prostaglandin I2 (prostacyclin) synthase	4.52969164	0.030765
*Fkbp10*	FK506 binding protein 10	4.43037992	0.039368
*Axin2*	Axin 2	4.416998695	0.022697
*Pla2g16*	Phospholipase A2, group XVI	4.319974957	0.039368
*Il17ra*	Interleukin 17 receptor A	3.86500417	0.031256
*Disp1*	Dispatched RND transporter family member 1	3.826121183	0.031256
*Ptgfrn*	Prostaglandin F2 receptor negative regulator	3.663983702	0.023473
*Greb1l*	Growth regulation by estrogen in breast cancer-like	3.513247405	0.0049
*Sptb*	Spectrin beta, erythrocytic	3.406728181	0.016367
*Aqp11*	Aquaporin 11	3.384139553	0.026891
*Extl3*	Exostoses (multiple)-like 3	3.381491933	0.037503
*Cttnbp2*	Cortactin binding protein 2	3.165960888	0.022697
*Kif21b*	Kinesin family member 21B	3.103400943	0.002999
*Fam213a*	Family with sequence similarity 213, member A	3.092980639	0.024278
*Auts2*	Autism susceptibility candidate 2	3.014691756	0.046838
*Tspan6*	Tetraspanin 6	2.872077594	0.046282
*Lrig1*	Leucine-rich repeats and immunoglobulin-like domains 1	2.738931683	0.046282
*Pi16*	Peptidase inhibitor 16	2.427155192	0.037503
*Laptm4b*	Lysosomal-associated protein transmembrane 4B	2.318212303	0.011483
*Slc7a6*	Solute carrier family 7 (cationic amino acid transporter, y+ system), member 6	2.267050692	0.024407
*Ptprg*	Protein tyrosine phosphatase, receptor type, G	2.158891183	0.022697
*Cachd1*	Cache domain containing 1	2.065569075	0.007219
*Rasgrp4*	RAS guanyl releasing protein 4	2.057762756	0.045899
*Klhl29*	Kelch-like 29	2.027131525	0.036831
*Bambi*	BMP and activin membrane-bound inhibitor	2.016141198	0.044925
*Mlec*	Malectin	2.003811198	0.022697
*Ppp1r9a*	Protein phosphatase 1, regulatory (inhibitor) subunit 9A	1.948922878	0.036956
*1810058I24Rik*	RIKEN cDNA 1810058I24 gene	1.899752884	0.038628
*Osbp2*	Oxysterol binding protein 2	1.86993046	0.024407
*Gm13889*	Predicted gene 13889	1.808734268	0.036956
*Vim*	Vimentin	1.808581233	0.041067
*Gnai1*	Guanine nucleotide binding protein (G protein), alpha inhibiting 1	1.795430532	0.036831
*Slc1a4*	Solute carrier family 1 (glutamate/neutral amino acid transporter), member 4	1.731445442	0.042161
*Cdc14a*	CDC14 cell division cycle 14A	1.727805031	0.041067
*Fbl*	Fibrillarin	1.67657139	0.045899
*Lmo2*	LIM domain only 2	1.650427031	0.043595
*1110051M20Rik*	RIKEN cDNA 1110051M20 gene	1.601538653	0.036831
*Cpt1c*	Carnitine palmitoyltransferase 1c	1.577681949	0.042456
*Fxyd5*	FXYD domain-containing ion transport regulator 5	1.552882564	0.038628
*Lrrc75a*	Leucine rich repeat containing 75A	1.496322239	0.046203
*Pgap1*	Post-GPI attachment to proteins 1	1.490320435	0.046282
*Zfp36l1*	Zinc finger protein 36, C3H type-like 1	1.474391469	0.037651
*Mir3092*	microRNA 3092	1.440648261	0.03784
*Ppic*	Peptidylprolyl isomerase C	1.435613585	0.043595
*Ppdpf*	Pancreatic progenitor cell differentiation and proliferation factor	1.336327784	0.041067
*Cdk4*	Cyclin-dependent kinase 4	1.32815528	0.037503
*St3gal4*	ST3 beta-galactoside alpha-2,3-sialyltransferase 4	1.281026042	0.036831
*D630024D03Rik*	RIKEN cDNA D630024D03 gene	1.238041514	0.037503
*Cdc45*	Cell division cycle 45	1.230144783	0.022697
*Eif4g1*	Eukaryotic translation initiation factor 4, gamma 1	1.143713408	0.043595
*Atp6v1d*	ATPase, H+ transporting, lysosomal V1 subunit D	0.893603982	0.048858
*Tmx3*	Thioredoxin-related transmembrane protein 3	0.84721687	0.048858
*Olfr1033*	Olfactory receptor 1033	0.83993199	0.042103
*Ergic2*	ERGIC and golgi 2	0.834146565	0.024407
*Aqp1*	Aquaporin 1	0.828501756	0.046731
*Glra2*	Glycine receptor, alpha 2 subunit	0.817240036	0.031256
*Gm26744*	Predicted gene, 26744	0.810903036	0.046282
*Spag9*	Sperm associated antigen 9	0.802558367	0.036831
*Rnf115*	Ring finger protein 115	0.80194744	0.036831
*Cd47*	CD47 antigen (Rh-related antigen, integrin-associated signal transducer)	0.800896378	0.031314
*Dpp4*	Dipeptidylpeptidase 4	0.794955116	0.041067
*Crebl2*	cAMP responsive element binding protein-like 2	0.788857661	0.022697
*Atp6ap2*	ATPase, H+ transporting, lysosomal accessory protein 2	0.782409663	0.024407
*Zfp763*	Zinc finger protein 763	0.779090085	0.007745
*Fez2*	Fasciculation and elongation protein zeta 2 (zygin II)	0.762992727	0.036831
*Zfp715*	Zinc finger protein 715	0.744750505	0.048858
*Cyb561d1*	Cytochrome b-561 domain containing 1	0.744262296	0.048858
*Extl2*	Exostoses (multiple)-like 2	0.728699546	0.048068
*Golga7*	Golgi autoantigen, golgin subfamily a, 7	0.723018512	0.026891
*Tgoln1*	Trans-golgi network protein	0.721857092	0.036831
*Tpm3*	Tropomyosin 3, gamma	0.717139012	0.044415
*Ggh*	Gamma-glutamyl hydrolase	0.714504325	0.048882
*Scrn3*	Secernin 3	0.707136697	0.046282
*Irak2*	Interleukin-1 receptor-associated kinase 2	0.68925238	0.037503
*Tmem170b*	Transmembrane protein 170B	0.685022667	0.046847
*Dgke*	Diacylglycerol kinase, epsilon	0.681193453	0.024407
*Itga1*	Integrin alpha 1	0.676125877	0.041067
*Sdccag8*	Serologically defined colon cancer antigen 8	0.662856645	0.041067
*Hspa12a*	Heat shock protein 12A	0.647460358	0.046907
*Nceh1*	Neutral cholesterol ester hydrolase 1	0.641787883	0.042161
*Impact*	Impact, RWD domain protein	0.641068766	0.022697
*Nlrc3*	NLR family, CARD domain containing 3	0.616870496	0.024407
*Pitpnm1*	Phosphatidylinositol transfer protein, membrane-associated 1	0.607880798	0.024407
*Gm19663*	Predicted gene, 19663	0.601230962	0.035201
*Inpp4b*	Inositol polyphosphate-4-phosphatase, type II	0.598629428	0.048227
*Pde3b*	Phosphodiesterase 3B, cGMP-inhibited	0.586483967	0.036956
*P2ry10b*	Purinergic receptor P2Y, G-protein coupled 10B	0.58265069	0.044415
*Ldhb*	Lactate dehydrogenase B	0.575509392	0.031256
*Smco4*	Single-pass membrane protein with coiled-coil domains 4	0.546281346	0.039368
*Gm14005*	Predicted gene 14005	0.545796227	0.046282
*A630072L19Rik*	RIKEN cDNA A630072L19 gene	0.545449977	0.037503
*Cyp7b1*	Cytochrome P450, family 7, subfamily b, polypeptide 1	0.537648924	0.039821
*Ldb2*	LIM domain binding 2	0.526662693	0.036831
*Nudt12*	Nudix (nucleoside diphosphate linked moiety X)-type motif 12	0.522324687	0.037503
*Cfh*	Complement component factor h	0.521077661	0.036831
*Ptpru*	Protein tyrosine phosphatase, receptor type, U	0.517441974	0.042879
*Pgghg*	Protein glucosylgalactosylhydroxylysine glucosidase	0.479186666	0.048882
*Acer3*	Alkaline ceramidase 3	0.472763137	0.036831
*Ceacam1*	Carcinoembryonic antigen-related cell adhesion molecule 1	0.472603157	0.041067
*Ccdc88c*	Coiled-coil domain containing 88C	0.469087339	0.027307
*Fam189a2*	Family with sequence similarity 189, member A2	0.467888229	0.041067
*Cysltr2*	Cysteinyl leukotriene receptor 2	0.442737637	0.023473
*Gramd1c*	GRAM domain containing 1C	0.42091094	0.004191
*Ntf3*	Neurotrophin 3	0.420199255	0.038876
*Fam174b*	Family with sequence similarity 174, member B	0.414689246	0.037572
*Slc26a10*	Solute carrier family 26, member 10	0.394412144	0.022697
*Pla2r1*	Phospholipase A2 receptor 1	0.354842083	0.048858
*Gpc1*	Glypican 1	0.354767031	0.022697
*Rnase4*	Ribonuclease, RNase A family 4	0.321464634	0.043595
*Olfm1*	Olfactomedin 1	0.276916317	0.036956
*Cd209b*	CD209b antigen	0.268887021	0.009718
*Flrt1*	Fibronectin leucine rich transmembrane protein 1	0.200652325	0.048858
*Ada*	Adenosine deaminase	0.153417209	0.022697

*Genes are displayed that were significantly up- or downregulated when compared to Ctnnb1^WT^ controls with Fold change (FC) < 1 or > 1. Adjusted p-values were calculated for the differences of means of log10 of expression values between Ctnnb1^OE−EC^ and Ctnnb1^WT^*.

An established panel of LSEC-associated and CEC-associated marker genes (Geraud et al., 2010, 2017; Olsavszky et al., 2020) was analyzed in LSECs isolated from *Ctnnb1*^*WT*^ control and *Ctnnb1*^*OE*−*EC*^ animals. Gene expression analysis pointed out that β-catenin GOF mutation in LSECs of *Ctnnb1*^*OE*−*EC*^ mice dysregulated LSEC- and CEC-associated genes (Figure 4C). GSEA revealed a significant induction of a CEC-associated gene set (Figure 4D) and a significant loss of an LSEC gene set (Figure 4E). As Wnt-/β-catenin signaling is a well-known driver for brain endothelial differentiation (Liebner et al., 2008), we hypothesized that β-catenin signaling activation in LSECs might result in partial brain EC reprograming. When performing GSEA with a brain endothelial gene set, which was generated by comparing published single-cell RNA-seq data from brain vs. liver ECs (Sabbagh et al., 2018), a significant enrichment for brain EC transcripts was found in *Ctnnb1*^*OE*−*EC*^ LSEC (Figure 4F). Among the genes that were significantly upregulated in *Ctnnb1*^*OE*−*EC*^ LSEC with a fold-change of >2, several genes could be detected that were also highly expressed in brain ECs (Table 2). As the expression of TJ molecule, Cldn5 was previously shown to be upregulated by endothelial Wnt-/β-catenin GOF in the leaky suprafornical organ (Benz et al., 2019), we compared the expression levels of Cldn5 in *Ctnnb1*^*OE*−*EC*^ and control liver. Expression of Cldn5 was not altered in *Ctnnb1*^*OE*−*EC*^ compared with control LSECs (Supplementary Figure 5A).

**Table 2:** Brain endothelial transcripts.

**Gene symbol**	**Gene title**	**Fold change** ***Ctnnb1***^***OE***^^**−*EC***^ **> *Ctnnb1***^***WT***^	**Adjusted p-value for Diff of genotype = ** ***Ctnnb1*** ^***OE***^ ^**−*EC***^ **–*Ctnnb1*** ^***WT***^
*Slc35f2*	Solute carrier family 35, member F2	35.13809	0.000397
*Myo1b*	Myosin IB	6.942870328	0.023473
*Tcf7*	Transcription factor 7, T cell specific	4.800597937	0.013887
*Axin2*	Axin 2	4.416998695	0.022697
*Pla2g16*	Phospholipase A2, group XVI	4.319974957	0.039368
*Il17ra*	Interleukin 17 receptor A	3.86500417	0.031256
*Extl3*	Exostoses (multiple)-like 3	3.381491933	0.037503
*Ptprg*	Protein tyrosine phosphatase, receptor type, G	2.158891183	0.022697
*Cachd1*	Cache domain containing 1	2.065569075	0.007219

*Genes are displayed that were significantly upregulated in Ctnnb1^OE−EC^ LSEC compared to Ctnnb1^WT^ LSEC with a fold change (FC) > 2 and also overexpressed in brain endothelial cells compared to liver endothelial cells (Daneman et al., 2010; Sabbagh et al., 2018)*.

**Figure 5:**
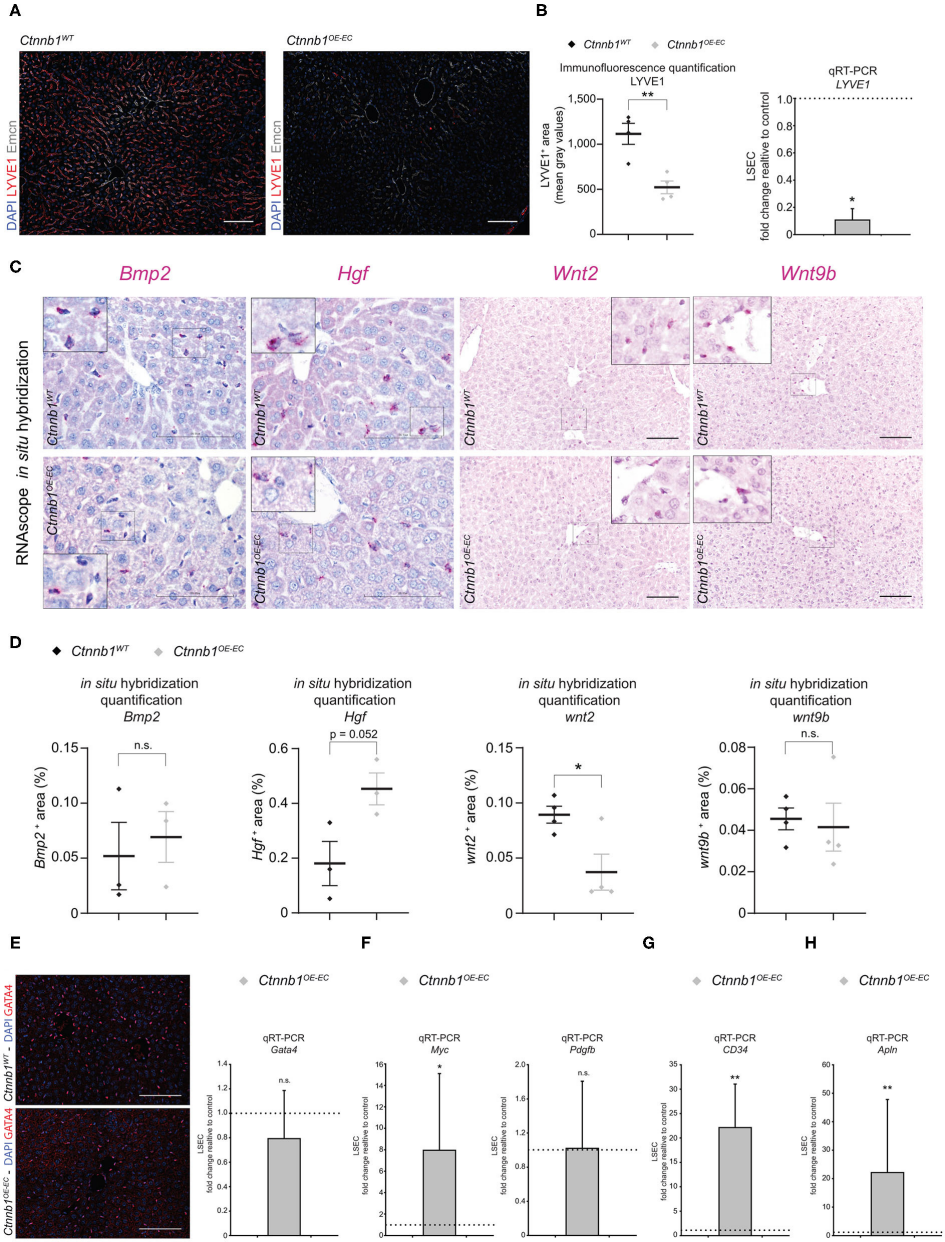
Hepatic endothelial *Ctnnb1* overactivation causes loss of LSEC-associated genes and induction of CEC and brain EC genes. **(A)** IF staining of DAPI, LYVE1, and Emcn in the liver of 2- to 3-month-old female *Ctnnb1*^*WT*^ and *Ctnnb1*^*OE*−*EC*^ mice (*n* = 4). Scale bar 100 μm. **(B)** Left panel: IF quantification of LYVE1^+^ area. Results are represented as mean ± SEM. ^**^p < 0.01. Right panel: qRT-PCR for *LYVE1* of cDNA from *Ctnnb1*^*OE*−*EC*^-LSECs compared to *Ctnnb1*^*WT*^ control LSECs (*n* = 4). β-Actin was used as housekeeping gene. ^*^p < 0.05. **(C)** *Bmp2, Hgf*, *Wnt2, Wnt9b* mRNA RNAScope *in situ* hybridization assay of 2- to 3-month-old female *Ctnnb1*^*WT*^ and *Ctnnb1*^*OE*−*EC*^ mice liver sections (*n* ≥ 3). Scale bar 100 μm. **(D)** Quantification of *Bmp2, Hgf*, *Wnt2, Wnt9b* mRNA RNAScope *in situ* hybridization assay. Results are represented as mean ± SEM. ns, not significant. ^*^p < 0.05. **(E)** Left panel: IF staining of DAPI and GATA4 in the liver of 2- to 3-month-old female *Ctnnb1*^*WT*^ and *Ctnnb1*^*OE*−*EC*^ mice (*n* = 4). Scale bar 100 μm. Right panel: qRT-PCR for *Gata4* with cDNA from *Ctnnb1*^*OE*−*EC*^-LSECs compared to *Ctnnb1*^*WT*^ control LSECs (*n* = 4). β-Actin was used as housekeeping gene. n.s., not significant. **(F)** qRT-PCR for *Myc* and *Pdgfb* with cDNA from *Ctnnb1*^*OE*−*EC*^-LSECs compared with *Ctnnb1*^*WT*^ control LSECs (*n* = 3). β-Actin was used as housekeeping gene. ^*^, *p* < 0.05; n.s., not significant. **(G)** qRT-PCR for *CD34* with cDNA from *Ctnnb1*^*OE*−*EC*^-LSECs compared with *Ctnnb1*^*WT*^ control LSECs (*n* = 3). β-Actin was used as housekeeping gene. ^**^p < 0.01. **(H)** qRT-PCR for *Apln* with cDNA from *Ctnnb1*^*OE*−*EC*^-LSECs compared with *Ctnnb1*^*WT*^ control LSECs (*n* = 3). β-Actin was used as housekeeping gene. ^**^p < 0.01.

In addition, the expression of markers for endothelial zonation, that is, Emcn and LYVE1 (Walter et al., 2014) were analyzed. A significant loss of mid-zonal LSEC marker LYVE1 was found, indicating disturbed endothelial liver zonation (Figures 5A,B). However, the expression of pericentral LSEC and CEC marker Emcn was not altered on protein level (Figure 5A). Despite disturbed endothelial zonation, the expression of pan-endothelial marker podocalyxin or CD31 was unaltered indicating no major changes in vascular density in *Ctnnb1*^*OE*−*EC*^ livers (Supplementary Figure 5B). Furthermore, β-catenin GOF in the LSECs did not alter the expression of LSEC marker CD32b (Supplementary Figure 5C), LSEC scavenger receptors *Stab1* and *Stab2* (Supplementary Figures 5D,E) or CEC markers *Vegfr2*, Caveolin-1, ICAM1, vascular cell adhesion molecule (VCAM), or VE-cadherin (Supplementary Figures 5C, 6A–C).

To confirm the transcriptomic alterations seen in *Ctnnb1*^*OE*−*EC*^ LSEC, we performed immunofluorescent staining, real-time quantitative PCR (qRT-PCR), and ISH for selected EC genes and proteins. The selection was either based on significant regulation among the list of CEC-associated genes (Figure 4C), relation to liver fibrosis [*Gata4, Myc*, platelet-derived growth factor subunit B (*Pdgfb*)] (Winkler et al., 2021), or established LSEC angiocrine factors. Upon ISH, the expression of the bone morphogenetic protein (*Bmp*) 2 was not significantly altered (Figures 5C,D). This was in line with Prussian blue staining of the liver, which did not show iron deposition in the liver of *Ctnnb1*^*OE*−*EC*^ mice (Supplementary Figure 4B). Moreover, *Hamp* expression in liver lysates was unaltered, indicating preserved BMP2–HAMP signaling (Supplementary Figure 4D). While LSEC angiocrine factor *Wnt2* was significantly downregulated, *Hgf* and *Wnt9b* were not significantly altered (Figures 5C,D). β-catenin GOF mutation in LSECs did not alter the expression of LSEC master regulator GATA4 on protein or mRNA level (Figure 5E) or pro-fibrotic angiocrine factor *Pdgfb* (Figure 5F). On the contrary, transcription factor *Myc* and CEC markers *CD34* and *Apln* were significantly upregulated in *Ctnnb1*^*OE*−*EC*^ LSEC (Figures 4C, 5F–H).

## Discussion

Our data show that imbalanced or overactivated β-catenin signaling in LSECs leads to sinusoidal transdifferentiation, including dysregulated lipid homeostasis. Reduced overall survival of *Ctnnb1*^*OE*−*EC*^ mice was most likely independent from LSEC transdifferentiation and dysregulated lipid homeostasis, but rather resulted from progressive heart dysfunction. The heart phenotype observed in *Ctnnb1*^*OE*−*EC*^ mice is comparable to β-catenin GOF mutation studies in arterial ECs by using a *Bmx-CreER*^T2^ mice, although reporter activity in heart ECs of *Clec4g-iCre;R26YFP* mice was identified in more than just arterial ECs, namely in heart capillaries, endocardium, and venous coronary vessels. Mechanistically, activation of Wnt-/β-catenin signaling in arterial ECs of the heart was shown to result in progressive heart failure through suppressing neuregulin-ErbB signaling (Nakagawa et al., 2016).

In the liver, β-catenin GOF mutation in LSECs resulted in sinusoidal-to-continuous transdifferentiation with downregulation of midzonal LSEC marker LYVE1 and angiocrine factor *Wnt2*, and upregulation of CEC markers *CD34* and *Apln*. This rather “mild” capillarization program lacking HSC activation and perisinusoidal extracellular matrix deposition did not result in hepatopathy or liver fibrosis. Interestingly, Wnt-target gene *Myc* (He et al., 1998) was significantly induced in *Ctnnb1*^*OE*−*EC*^ LSECs. Previous work by us could show, that loss of LSEC master regulator GATA4 also induced pro-angiogenic *Myc* in LSECs, to further amplify a pro-fibrotic angiocrine program, including *de novo Pdgfb* expression, resulting in perisinusoidal liver fibrosis (Winkler et al., 2021). β-catenin GOF in LSECs did not significantly regulate GATA4 expression, which most likely protects against a complete capillarization program and perisinusoidal liver fibrosis by suppressing pro-fibrotic angiocrine factors such as *Pdgfb*, which was unaltered in *Ctnnb1*^*OE*−*EC*^ LSEC despite a significant *Myc* induction.

Angiocrine Wnt-signaling is vital for liver growth and metabolic liver zonation and Wnt-signaling in LSECs is linked to autocrine growth effects (Klein et al., 2008; Geraud et al., 2010; Leibing et al., 2018). While activation of β-catenin in LSECs reduced angiocrine *Wnt2*, this reduction together with unaltered *Wnt9b* was not sufficient to impair metabolic liver zonation in *Ctnnb1*^*OE*−*EC*^ mice. Interestingly, EC proliferation was significantly induced by activation of β-catenin in LSECs. These findings are supported by GSEA results of *Ctnnb1*^*OE*−*EC*^ LSEC with enrichment in the gene sets “G2M Checkpoint” and “E2F Targets,” both resembling a pro-proliferative state, thereby indicating that β-catenin overactivation in LSECs stimulates endothelial proliferation. This is in line with data observed in postnatal brain and retina, showing that deficiency of endothelial β-catenin signaling impairs endothelial proliferation and sprouting (Martowicz et al., 2019).

Notably, activation of β-catenin in LSECs resulted in the upregulation of genes that are known to be expressed by the brain ECs (Daneman et al., 2010; Wang et al., 2019) and GSEA could confirm the enrichment of brain EC transcripts (Sabbagh et al., 2018) in *Ctnnb1*^*OE*−*EC*^ LSECs. In contrast to LSEC, that belong to discontinuous sinusoidal ECs which enable transfer of fluids, nutrients, and small solutes through open fenestrations within the sinusoidal wall (Wisse et al., 1985; Augustin and Koh, 2017), the brain ECs belong to the group of CECs, expressing specialized TJ molecules and transporters for restricting paracellular passage and transcellular trafficking, thereby generating the tightly sealed blood–brain barrier (Langen et al., 2019). In line with our results, ectopic β-catenin signaling activation in the highly permeable and fenestrated vasculature of the circumventricular organs is sufficient for BBB reprograming (Benz et al., 2019; Wang et al., 2019). Furthermore, inducible pan-endothelial Ctnnb1 GOF showed some overlap with genes dysregulated in *Ctnnb1*^*OE*−*EC*^ LSEC despite using different Cre lines (Munji et al., 2019). Vice-versa, loss of Wnt-signaling activity impairs brain endothelial differentiation by downregulating TJ molecules and transporter proteins, while increasing the expression of the plasmalemma vesicle–associated protein (PLVAP) (Liebner et al., 2008; Stenman et al., 2008; Daneman et al., 2009). However, the expression of TJ molecule Cldn5 was not enhanced in *Ctnnb1*^*OE*−*EC*^ LSEC, which could be a result of maintained expression of LSEC master regulator GATA4. Notably, PLVAP knockout mice developed a reduction of LSEC fenestrations, which led to elevated serum levels of triglycerides, low-density lipoprotein, and cholesterol due to retention of chylomicron remnants in the blood. The authors speculated that compensatory hepatocyte *de novo* lipogenesis was responsible for steatosis, steatohepatitis, and liver fibrosis (Herrnberger et al., 2014). *Ctnnb1*^*OE*−*EC*^ mice neither showed liver steatosis nor fibrosis, which argue against reduced PLVAP expression as a main driver of isolated hypertriglyceridemia in *Ctnnb1*^*OE*−*EC*^ mice.

As only microvascular ECs in the liver with sinusoidal differentiation allow filtration of chylomicron remnants from the blood (Fraser et al., 1995; Cogger et al., 2006), β-catenin-mediated transdifferentiation of liver sinusoids with partial BBB reprograming in *Ctnnb1*^*OE*−*EC*^ mice may impair uptake of chylomicrons and subsequently lead to elevated serum lipid levels. However, these metabolic alterations are in contrast with previous results, showing that neither sinusoidal capillarization with loss of fenestrations and formation of a basement membrane in Gata4-deficient LSEC (*Gata4*^*LSEC*−*KO*^), nor partial sinusoidal capillarization/trandifferentiation in mice with enhanced Notch signaling in LSECs (*NICD*^*OE*−*HEC*^) are associated with reduced levels of serum cholesterol and triglycerides (Wohlfeil et al., 2019; Winkler et al., 2021). This argues against a general impairment of lipid transfer into the space of Disse during sinusoidal capillarization/transdifferentiation and indicates that hypertriglyceridemia in *Ctnnb1*^*OE*−*EC*^ mice is a result of β-catenin-mediated LSEC transdifferentiation by impaired transendothelial transport mechanisms and/or by altered angiocrine signaling that control hepatocyte lipogenesis/lipolysis.

Among the *de novo* expressed genes in *Ctnnb1*^*OE*−*EC*^ LSEC, *Apln* was found as the second most upregulated gene. Apelin (APLN) is a secreted peptide, which is widely expressed in different cell types, including CECs and is also known as a regulator of transendothelial lipid transport (Hwangbo et al., 2017). Mice with *Apln* knockout become obese and show more fat deposition as a consequence of increased vascular permeability with greater uptake of fatty acids. On the other hand, transgenic *Apln* mice that express apelin under the transcriptional control of the keratin 14 promoter are protected from obesity and show a reduced endothelial permeability (Sawane et al., 2011, 2013). Interestingly, Huang and colleagues were able to show that also Apln signaling in hepatocytes protects against lipid accumulation in the liver (Huang et al., 2017). As the promoter region of the *Apln* gene has transcription factor–binding sites for Wnt signaling downstream targets *Tcf/Lef* (Chen et al., 2019), *Apln* expression might be transcriptionally activated by Wnt-β-catenin signaling activation in *Ctnnb1*^*OE*−*EC*^ LSECs. This is in line with silencing experiments of β-catenin in pulmonary ECs showing that *Apln* mRNA and protein expression were reduced (Alastalo et al., 2011). Thus, in *Ctnnb1*^*OE*−*EC*^ mice *de novo Apln* expression in transdifferentiated LSECs may be involved in dysregulated lipid homeostasis. Yet, one has to consider that aberrant *Apln* expression is also found in CD34^+^ capillarized LSECs in liver fibrosis (Winkler et al., 2021) and cirrhosis (Yokomori et al., 2012) and also pro-angiogenic effects were similar to the vascular apelin signaling (Helker et al., 2020).

Together, normal sinusoidal differentiation is decisive for the fulfillment of the typical LSEC functions such as scavenging, immunoregulation, protection against stellate cell activation, and fibrosis, but also for the angiocrine regulation of liver regeneration and iron metabolism (Poisson et al., 2017; Shetty et al., 2018; Lafoz et al., 2020; Koch et al., 2021). While endothelial Wnt-signaling activity is largely confined to brain ECs for the maintenance of the BBB (Sabbagh et al., 2018), here we can show for the first time that low-level liver endothelial Wnt-signaling *in vivo* is crucial for maintaining sinusoidal differentiation, which is required for regulation of proper hepatic lipid metabolism. Further research is necessary to analyze the specific contributions of LSECs in hepatic fat absorption and metabolism. Future work will have to address which angiocrine signaling pathways may be involved in this process, extending the knowledge that liver endothelial fatty acid absorption is not mainly a passive mechanism mediated by open fenestrations in LSECs. This is of particular interest as dyslipidemia is a major risk factor for cardiovascular disease.

## Data Availability Statement

The datasets presented in this study can be found in online repositories. The names of the repository/repositories and accession number(s) can be found at: https://www.ncbi.nlm.nih.gov/geo/query/acc.cgi?acc=GSE175777.

## Ethics Statement

The animal study was reviewed and approved by Regional Council Karlsruhe. Written informed consent was obtained from the owners for the participation of their animals in this study.

## Author Contributions

P-SK, KSa, SG, and VO: study concept and design. P-SK, KSa, JHeil, CDS, SK, JHo, MW, CT, CS, KSc, MT, FT, JHein, CG, SG, and VO: experimental work, analysis, and interpretation of data. P-SK, KSa, and VO: writing original draft. All authors writing and reviewing the manuscript before submission.

## Funding

This work was supported by the Deutsche Forschungsgemeinschaft (DFG, German Research Foundation)—Project number 259332240—RTG 2099 (to CG, SG, and P-SK), Project number 5454871—SFB TR23 (to CG and SG), Project number 394046768—SFB 1366 (to CG, SG, and P-SK), Project number 413262200—ICON/EB 187/8-1 and Project number 314905040—CRC/SFB-TR 209 (to SG). The authors gratefully acknowledge the data storage service SDS@hd supported by the Ministry of Science, Research and the Arts Baden-Württemberg (MWK) and the German Research Foundation (DFG) through grant INST 35/1314-1 FUGG and INST 35/1503-1 FUGG. The funders had no role in study design, data collection and analysis, decision to publish, or preparation of the manuscript.

## Conflict of Interest

The authors declare that the research was conducted in the absence of any commercial or financial relationships that could be construed as a potential conflict of interest.

## Publisher's Note

All claims expressed in this article are solely those of the authors and do not necessarily represent those of their affiliated organizations, or those of the publisher, the editors and the reviewers. Any product that may be evaluated in this article, or claim that may be made by its manufacturer, is not guaranteed or endorsed by the publisher.
