# Supplementary material for: Imbalanced Activation of Wnt-/β-Catenin-Signaling in Liver Endothelium Alters Normal Sinusoidal Differentiation
Source: Front Physiol. 2021 Sep 29;12:722394. doi: 10.3389/fphys.2021.722394 (PMC8511684; doi:10.3389/fphys.2021.722394)
Supplement: Supplementary file 1 [file Data_Sheet_1.pdf]

Supplementary Material

1. Supplementary Figure Legends

Supplementary Figure 1. *Clec4g-iCre;R26YFP* mice show a pan-endothelial reporter activity in the heart. Immunofluorescence (IF) staining for endothelial markers of *Clec4g-iCre;R26YFP* hearts. **(A)** Co-IF of YFP, DAPI, CD31 and smooth muscle alpha-actin (αSMA) shown as single-channel and merged photomicrographs (n = 3). Scale bars: 100 µm. **(B)** Co-IF of YFP, DAPI, Emcn and LYVE1 shown as single-channel and merged photomicrographs (n = 3). Scale bars: 100 µm.

Supplementary Figure 2. *Ctnnb1*$^{OE\text{-}EC}$ mice display a progressive dilated cardiomyopathy. (A) Left ventricle (LV) volume, **(B)** LV posterior wall thickness, **(C)** LV anterior wall thickness, and **(D)** cardiac output as determined by echocardiography of 2, 4 and 6 weeks old *Ctnnb1*^{WT} and *Ctnnb1*$^{OE\text{-}EC}$ mice ($n \geq 3$). Results are represented as mean ± S.E.M.. *, $p < 0.05$; **, $p < 0.01$; ***, $p < 0.001$.

Supplementary Figure 3. No major differences in H&E stainings of internal organs of *Ctnnb1*$^{OE\text{-}EC}$ mice. H&E staining for sections from kidney, lung, spleen, brain (scale bar: 100 µm) and intestine (scale bar: 500 µm) of 2-3 months old female *Ctnnb1*^{WT} and *Ctnnb1*$^{OE\text{-}EC}$ mice (n = 3).

Supplementary Figure 4. Livers of *Ctnnb1*$^{OE\text{-}EC}$ mice do not show major differences in routine stainings or alterations in metabolic zonation and perisinusoidal collagen deposition. (A) Co-IF staining of DAPI, Collagen I (Col I), Collagen III (Col III) and Collagen IV (Col IV) in the liver of 2-3 months old female *Ctnnb1*^{WT} and *Ctnnb1*$^{OE\text{-}EC}$ mice (n = 4). Scale bar 100 µm. **(B)** H&E, PAS and Prussian blue staining of liver sections from 2-3 months old female *Ctnnb1*^{WT} and *Ctnnb1*$^{OE\text{-}EC}$ mice (n = 3). **(C)** Co-IF of DAPI, Emcn and CypeE1 in the liver of 3 months old female *Ctnnb1*^{WT} and *Ctnnb1*$^{OE\text{-}EC}$ mice (n = 4). Scale bars: 100 µm. **(D)** qRT-PCR for *Hamp* of cDNA from whole liver

lysates of 3 months old female *Ctnnb1*$^{OE\text{-}EC}$ mice compared to corresponding *Ctnnb1*^{WT} controls (n = 3). β-Actin was used as housekeeping gene. n.s., not significant.

Supplementary Figure 5. No major differences in selected LSEC-associated markers in livers, as well as in the hepatic vascular density of *Ctnnb1*$^{OE\text{-}EC}$ mice. (A) Co-IF and quantification of DAPI, Emcn and Cldn5 in the liver of 3 months old female *Ctnnb1*^{WT} and *Ctnnb1*$^{OE\text{-}EC}$ mice (n = 4). Scale bars: 100 µm. Results are represented as mean ± S.E.M.. n.s., not significant. **(B)** Co-IF of DAPI, Podocalyxin (Podxl) and CD31 and quantification of Podxl and CD31 in the liver of 3 months old female *Ctnnb1*^{WT} and *Ctnnb1*$^{OE\text{-}EC}$ mice (n = 5). Scale bars: 100 µm. Results are represented as mean ± S.E.M.. n.s., not significant. **(C)** Co-IF of DAPI, CD32b and Cav1 in the liver of 3 months old female *Ctnnb1*^{WT} and *Ctnnb1*$^{OE\text{-}EC}$ mice (n = 4). Scale bars: 100 µm. **(D)** *Stab1* and *Stab2* mRNA RNAScope® *in situ* hybridization assay of 3 months old female *Ctnnb1*^{WT} and *Ctnnb1*$^{OE\text{-}EC}$ mice liver sections (n = 4). Scale bar 50 µm. **(E)** qRT-PCR for *Stab1* and *Stab2* of cDNA from freshly isolated LSECs of 2 months old *Ctnnb1*$^{OE\text{-}EC}$ mice compared to corresponding *Ctnnb1*^{WT} controls (n = 3). β-Actin was used as housekeeping gene. n.s., not significant.

Supplementary Figure 6. No major differences in selected CEC-associated markers in livers from *Ctnnb1*$^{OE\text{-}EC}$ mice. (A) qRT-PCR for *KDR/VEGFR2* and *Cav1* of cDNA from freshly isolated LSECs of 2 months old *Ctnnb1*$^{OE\text{-}EC}$ mice compared to corresponding *Ctnnb1*^{WT} controls (n = 3). β-Actin was used as housekeeping gene. n.s., not significant. **(B)** Co- IF staining of DAPI, CD31, ICAM1, VCAM and VE-Cadherin in the liver of 2-3 months old female *Ctnnb1*^{WT} and *Ctnnb1*$^{OE\text{-}EC}$ mice (n = 4). Scale bar 100 µm.

2. Supplementary Figures

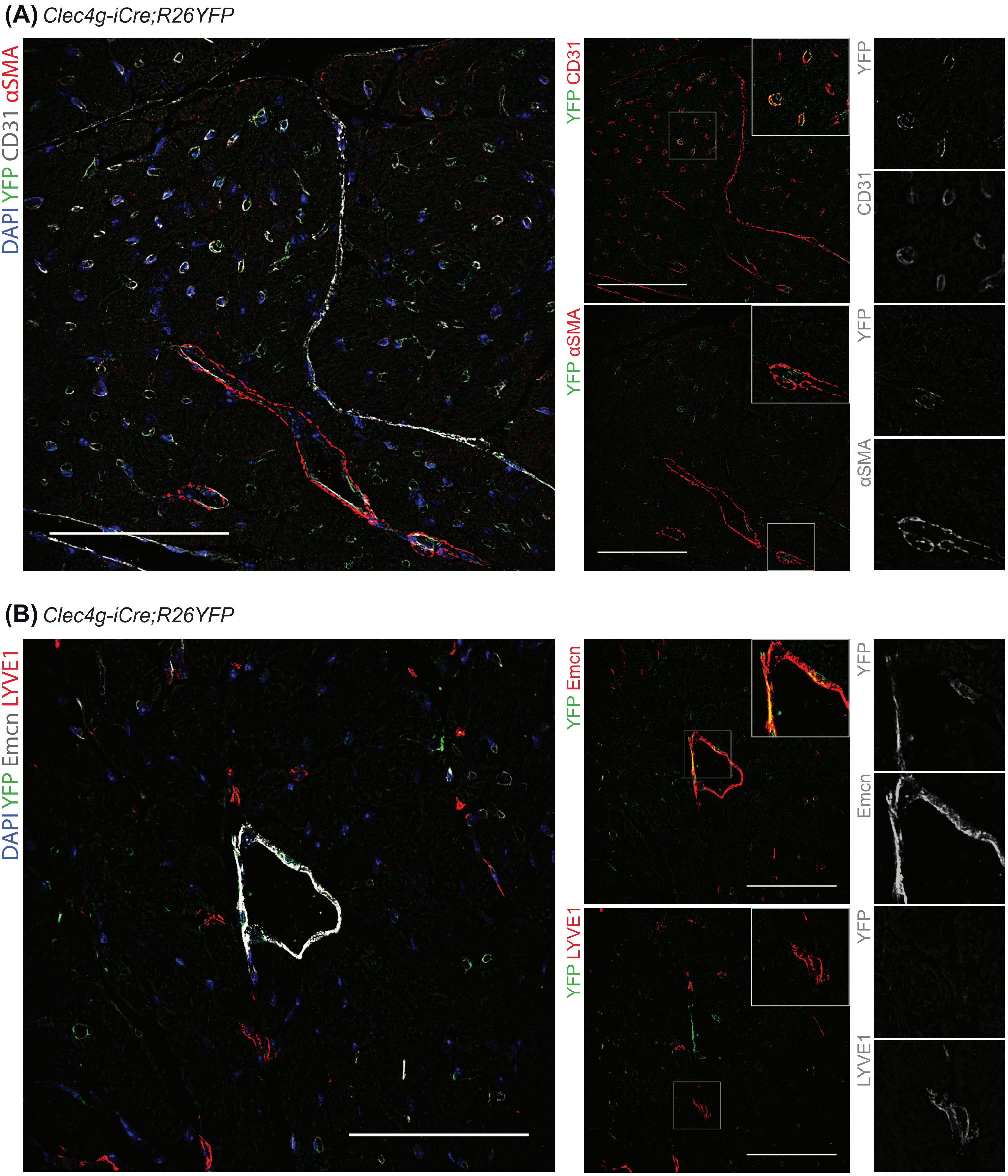
(A) Clec4g-iCre;R26YFP
DAPI YFP CD31 αSMA
YFP CD31
YFP
CD31
YFP αSMA
YFP
αSMA
(B) Clec4g-iCre;R26YFP
DAPI YFP Emcn LYVE1
YFP Emcn
YFP
Emcn
YFP LYVE1
YFP
LYVE1

Supp. Fig. 1.

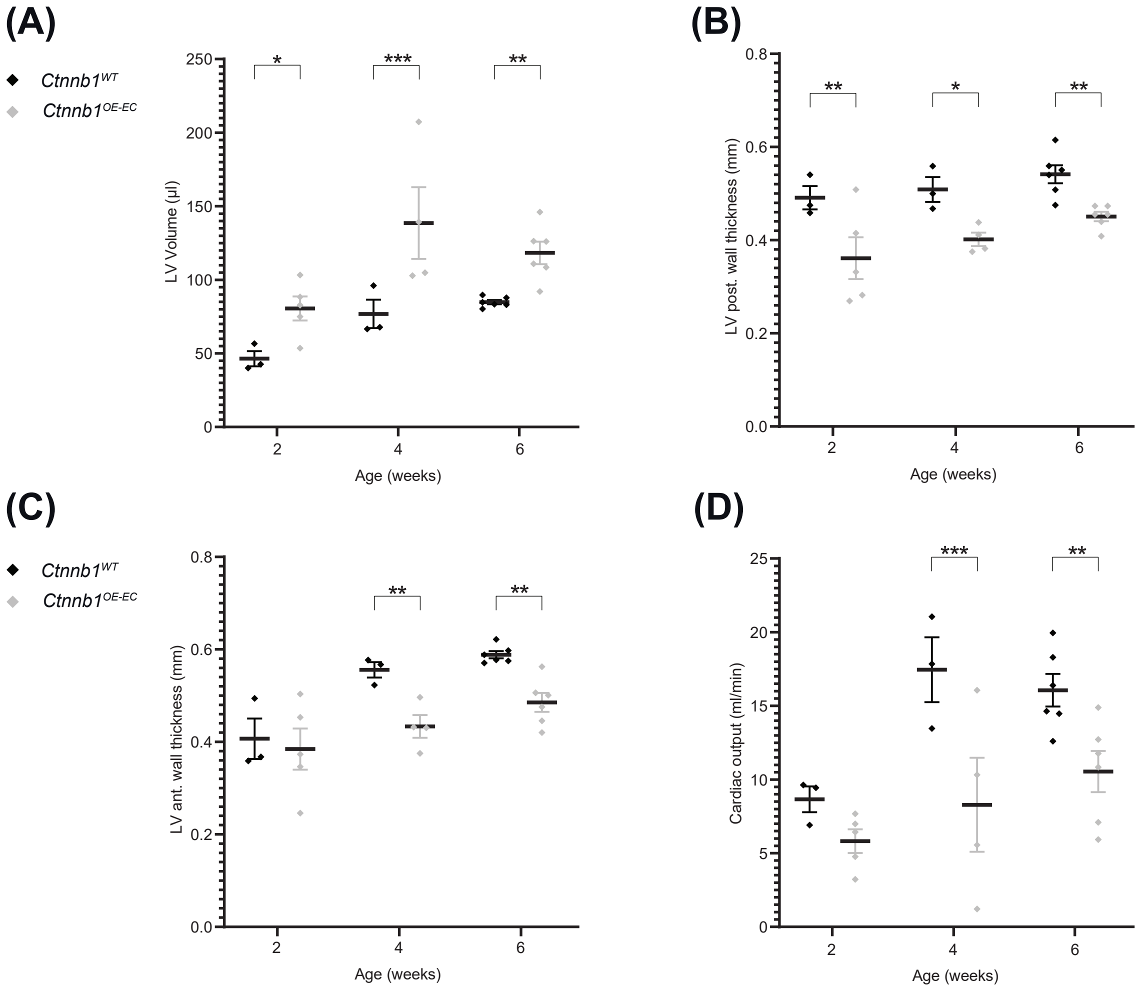

(A)
Ctnnb1WT
Ctnnb1OE-EC
LV Volume (µl)
Age (weeks)
(B)
LV post. wall thickness (mm)
Age (weeks)
(C)
Ctnnb1WT
Ctnnb1OE-EC
LV ant. wall thickness (mm)
Age (weeks)
(D)
Cardiac output (ml/min)
Age (weeks)

Supp. Fig. 2.

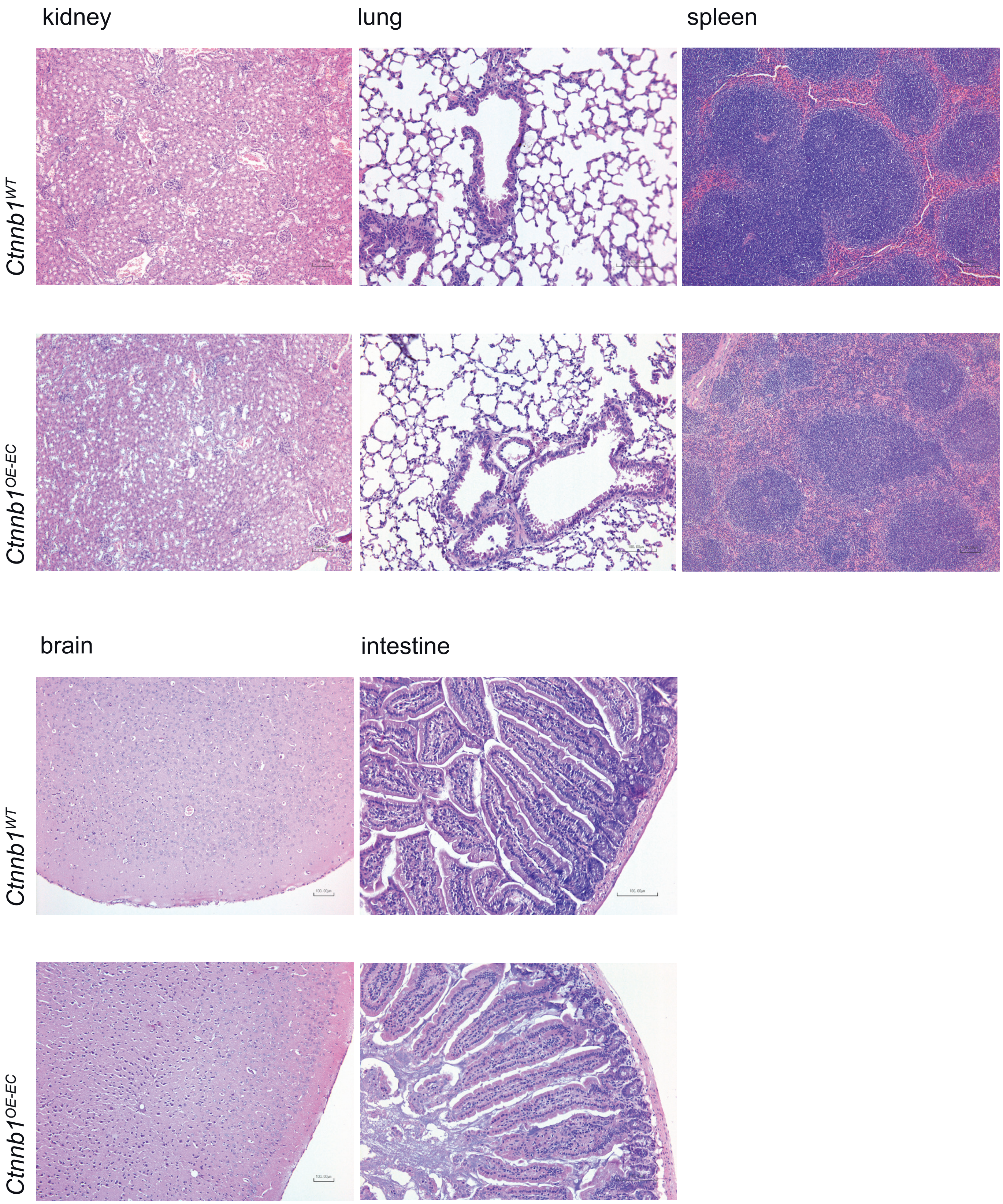

kidney
lung
spleen
brain
intestine
$Ctnnb1^{WT}$
$Ctnnb1^{OE\text{-}EC}$
$Ctnnb1^{WT}$
$Ctnnb1^{OE\text{-}EC}$

Supp. Fig. 3.

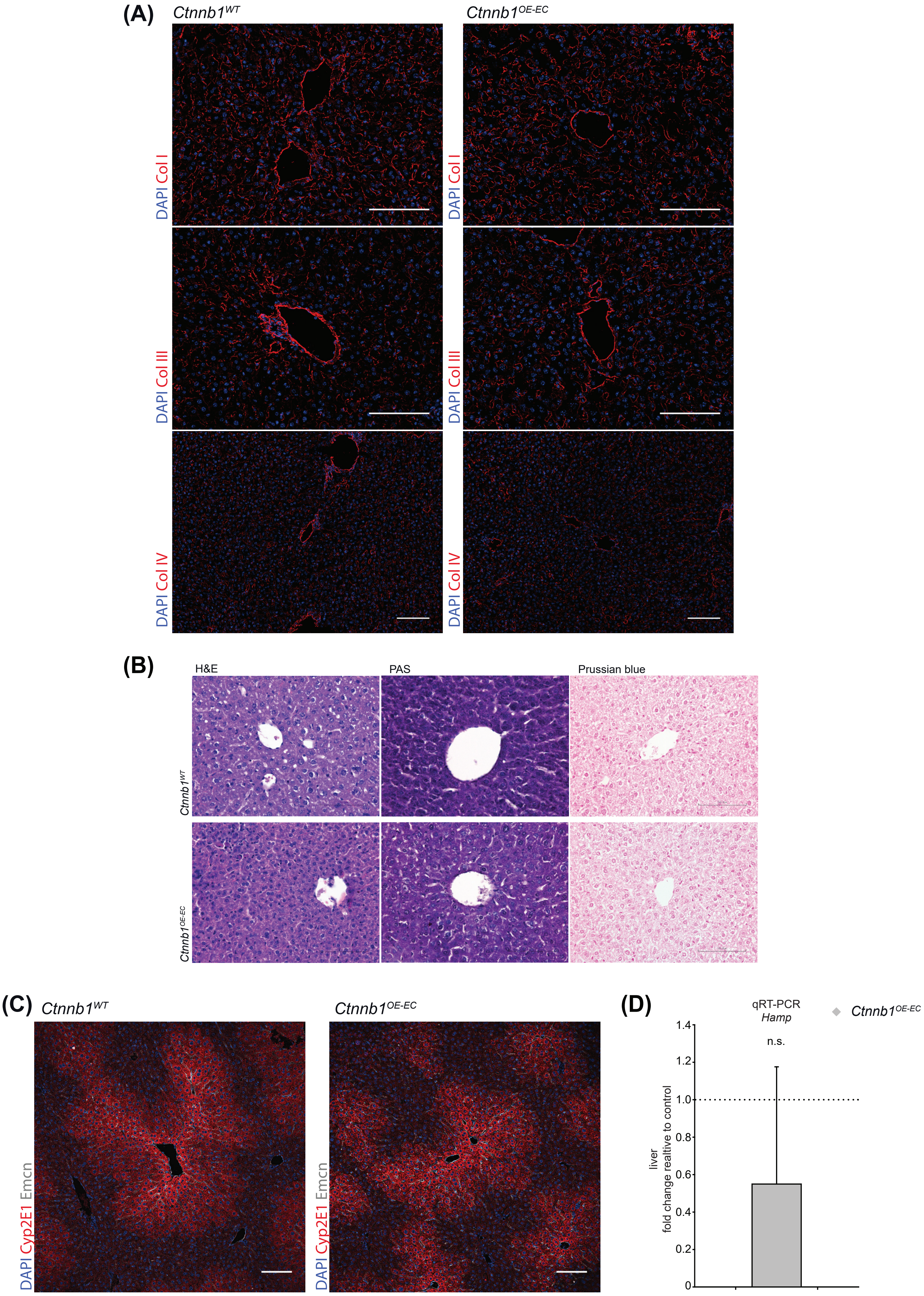
(A)
Ctnnb1WT
Ctnnb1OE-EC
DAPI Col I
DAPI Col III
DAPI Col IV
(B)
H&E
PAS
Prussian blue
Ctnnb1WT
Ctnnb1OE-EC
(C)
Ctnnb1WT
Ctnnb1OE-EC
DAPI Cyp2E1 Emcn
(D)
qRT-PCR
Hamp
Ctnnb1OE-EC
n.s.
liver
fold change realtive to control
1.4
1.2
1.0
0.8
0.6
0.4
0.2
0

Supp. Fig. 4.

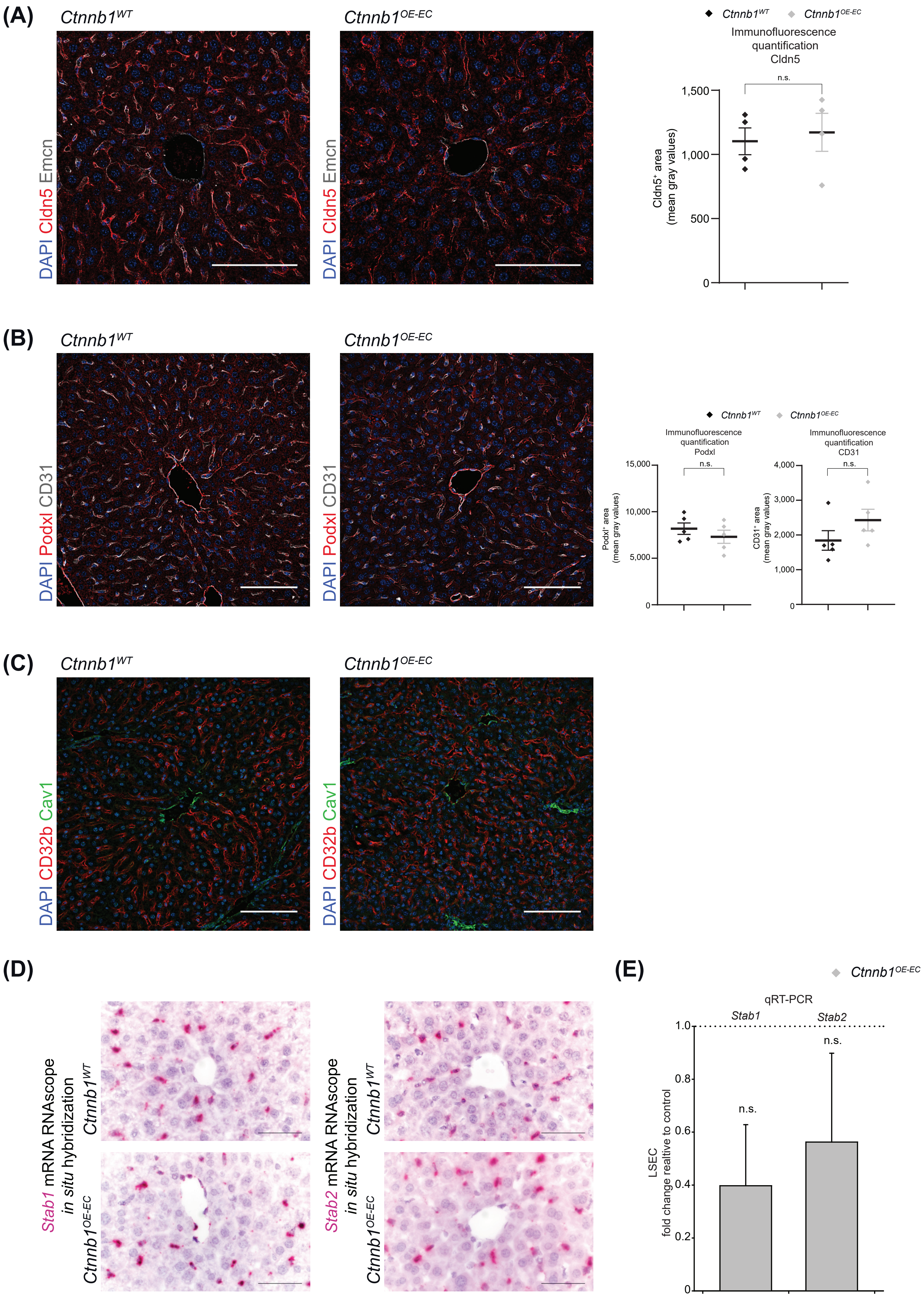
(A)
Ctnnb1WT
Ctnnb1OE-EC
DAPI Cldn5 Emcn
Ctnnb1WT
Ctnnb1OE-EC
Immunofluorescence quantification Cldn5
n.s.
1,500
1,000
500
0
Cldn5+ area (mean gray values)
(B)
Ctnnb1WT
Ctnnb1OE-EC
DAPI Podxl CD31
Immunofluorescence quantification Podxl
Immunofluorescence quantification CD31
n.s.
15,000
10,000
5,000
0
Podxl+ area (mean gray values)
4,000
3,000
2,000
1,000
0
CD31+ area (mean gray values)
(C)
Ctnnb1WT
Ctnnb1OE-EC
DAPI CD32b Cav1
(D)
Stab1 mRNA RNAscope in situ hybridization
Stab2 mRNA RNAscope in situ hybridization
Ctnnb1WT
Ctnnb1OE-EC
(E)
Ctnnb1OE-EC
qRT-PCR
Stab1
Stab2
n.s.
1.0
0.8
0.6
0.4
0.2
0
LSEC fold change realtive to control

Supp. Fig. 5.

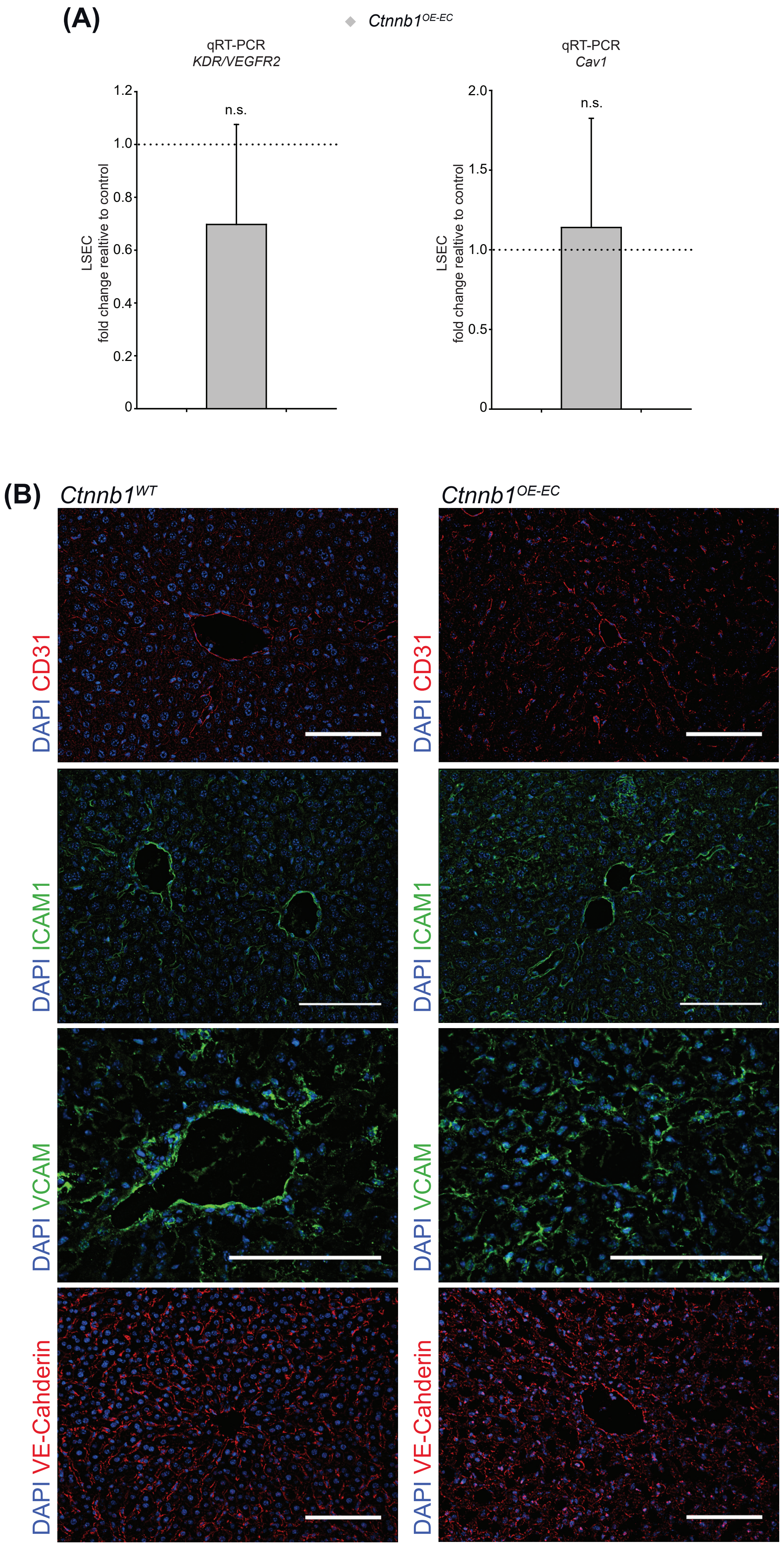
(A)
Ctnnb1OE-EC
qRT-PCR
KDR/VEGFR2
n.s.
LSEC
fold change realtive to control
1.2
1.0
0.8
0.6
0.4
0.2
0
qRT-PCR
Cav1
n.s.
2.0
1.5
1.0
0.5
0
(B)
Ctnnb1WT
Ctnnb1OE-EC
DAPI CD31
DAPI ICAM1
DAPI VCAM
DAPI VE-Cahderin

Supp. Fig. 6.
